# Findings from the Quebec Family Study on the Etiology of Obesity: Genetics and Environmental Highlights

**DOI:** 10.1007/s13679-013-0086-3

**Published:** 2014-01-04

**Authors:** Jean-Philippe Chaput, Louis Pérusse, Jean-Pierre Després, Angelo Tremblay, Claude Bouchard

**Affiliations:** 1 Healthy Active Living and Obesity Research Group, Children’s Hospital of Eastern Ontario Research Institute, 401 Smyth Road, Ottawa, ON K1H 8L1 Canada; 2 Department of Kinesiology, Faculty of Medicine, Laval University, 2300 de la Terrasse, Quebec City, QC G1V 0A6 Canada; 3 Centre de Recherche de l’Institut Universitaire de Cardiologie et de Pneumologie de Québec, Hôpital Laval, 2725 Chemin Sainte-Foy, Quebec City, QC G1V 4G5 Canada; 4 Human Genomics Laboratory, Pennington Biomedical Research Center, 6400 Perkins Road, Baton Rouge, LA 70808-4124 USA

**Keywords:** Genes, Environment, Physical activity, Diet, Nutrition, Eating behavior, Sleep, Calcium, Cohort, Observational study, Longitudinal study, Quebec Family Study, Obesity

## Abstract

The Quebec Family Study (QFS) was an observational study with three cycles of data collection between 1979 and 2002 in Quebec City, Canada. The cohort is a mixture of random sampling and ascertainment through obese individuals. The study has significantly contributed to our understanding of the determinants of obesity and associated disease risk over the past 35 years. In particular, the QFS cohort was used to investigate the contribution of familial resemblance and genetic effects on body fatness and behaviors related to energy balance. Significant familial aggregation and genetic heritability were reported for total adiposity, fat-free mass, subcutaneous fat distribution, abdominal and visceral fat, resting metabolic rate, physical activity level and other behavioral traits. The resources of QFS were also used to study the contribution of several nontraditional (non-caloric) risk factors as predictors of excess body weight and gains in weight and adiposity over time, including low calcium and micronutrient intake, high disinhibition eating behavior trait, and short sleep duration. An important finding relates to the interactions between dietary macronutrient intake and exercise intensity on body mass and adiposity.

**Electronic supplementary material:**

The online version of this article (doi:10.1007/s13679-013-0086-3) contains supplementary material, which is available to authorized users.

## Introduction

The main goal of the Quebec Family Study (QFS) as originally planned was to investigate the role of genetic factors in physical fitness, body composition, risk factors for common diseases and health related behavior. So far, 276 papers have been published totally or partly based on the QFS data and these papers have been cited more than 12,000 times for an average of about 43 citations per paper (see Table 1 for the top 25 articles in terms of Citations and Supplementary material for the complete list of QFS publications). The cross-sectional and longitudinal analytical design segments of QFS have been instrumental in the investigation of the etiology of obesity from a genetic and behavioral standpoint. The objective of the present review is to highlight some of the genetic and behavioral findings with regards to body weight, adiposity, adipose tissue distribution and obesity.

**Table 1:** The 25 papers based on the Quebec Family Study with the highest number of citations as of September 2013

Rank	Article	Number of Citations
1	Pouliot MC, Després JP, Lemieux S, Moorjani S, Bouchard C, Tremblay A, Nadeau A, Lupien PJ. Waist circumference and abdominal sagittal diameter: best simple anthropometric indexes of abdominal visceral adipose tissue accumulation and related cardiovascular risk in men and women. Am J Cardiol 1994;73:460–8.	955
2	Bouchard C, Tremblay A, Leblanc C, Lortie G, Savard R, Thériault G. A method to assess energy expenditure in children and adults. Am J Clin Nutr 1983;37:461–7.	431
3	Lemieux S, Prud’homme D, Bouchard C, Tremblay A, Després JP. A single threshold value of waist girth identifies normal-weight and overweight subjects with excess visceral adipose tissue. Am J Clin Nutr 1996;64:685–93.	258
4	Bouchard C, Pérusse L, Leblanc C, Tremblay A, Thériault G. Inheritance of the amount and distribution of human body fat. Int J Obes 1988;12:205–15.	255
5	Seidell JC, Pérusse L, Després JP, Bouchard C. Waist and hip circumferences have independent and opposite effects on cardiovascular disease risk factors: the Quebec Family Study. Am J Clin Nutr 2001;74:315–21.	230
6	Tremblay A, Plourde G, Després JP, Bouchard C. Impact of dietary fat content and fat oxidation on energy intake in humans. Am J Clin Nutr 1989;49:799–805.	224
7	Bouchard C, Pérusse L, Chagnon YC, Warden C, Ricquier D. Linkage between markers in the vicinity of the uncoupling protein 2 gene and resting metabolic rate in humans. Hum Mol Genet 1997;6:1887–9.	209
8	Lemieux S, Prud’homme D, Bouchard C, Tremblay A, Després JP. Sex differences in the relation of visceral adipose tissue accumulation to total body fatness. Am J Clin Nutr 1993;58:463–7.	206
9	Pérusse L, Tremblay A, Leblanc C, Bouchard C. Genetic and environmental influences on level of habitual physical activity and exercise participation. Am J Epidemiol 1989;129:1012–22.	195
10	Després JP, Allard C, Tremblay A, Talbot J, Bouchard C. Evidence for a regional component of body fatness in the association with serum lipids in men and women. Metabolism 1985;34:967–73.	194
11	Rice T, Rankinen T, Province MA, Chagnon YC, Pérusse L, Borecki IB, Bouchard C, Rao DC. Genome-Wide Linkage Analysis of Systolic and Diastolic Blood Pressure, The Quebec Family Study. Circulation 2000;102:1956–63.	178
12	Lembertas AV, Pérusse L, Chagnon YC, Fisler JS, Warden CH, Purcell-Huynh DA, Dionne FT, Gagnon J, Nadeau A, Lusis AJ, Bouchard C. Identification of an obesity quantitative trait locus on mouse chromosome 2 and evidence of linkage to body fat and insulin on the human homologous region 20q. J Clin Invest 1997;100:1240–7.	169
13	Rankinen T, Kim SY, Pérusse L, Després JP, Bouchard C. The prediction of abdominal visceral fat level from body composition and anthropometry: ROC analysis. Int J Obes 1999;23:801–9.	163
14	Oppert JM, Vohl MC, Chagnon M, Dionne FT, Cassard-Doulcier AM, Ricquier D, Pérusse L, Bouchard C. DNA polymorphism in the uncoupling protein (UCP) gene and human body fat. Int J Obes 1994;18:526–31.	154
15	Rosmond R, Chagnon YC, Holm G, Chagnon M, Pérusse L, Lindell K, Carlsson B, Bouchard C, Bjorntorp P. A Glucocorticoid Receptor Gene Marker is Associated with Abdominal Obesity, Leptin, and Dysregulation of the Hypothalamic-Pituitary-Adrenal Axis. Obes Res 2000;8:211–8.	150
16	Jacqmain M, Doucet E, Després JP, Bouchard C, Tremblay A. Calcium intake, body composition, and lipoprotein-lipid concentrations in adults. Am J Clin Nutr 2003;77:1448–52.	147
17	Buemann B, Vohl MC, Chagnon M, Chagnon YC, Gagnon J, Pérusse L, Dionne F, Després JP, Tremblay A, Nadeau A, Bouchard C. Abdominal visceral fat is associated with a BclI restriction fragment length polymorphism at the glucocorticoid receptor gene locus. Obes Res 1997;5:186–92.	140
18	Chaput JP, Després JP, Bouchard C, Tremblay A. Short sleep duration is associated with reduced leptin levels and increased adiposity: results from the Quebec Family Study. Obesity 2007;15:253–61.	123
19	Spiegelman D, Israel RG, Bouchard C, Willett WC. Absolute fat mass, percent body fat, and body-fat distribution: which is the real determinant of blood pressure and serum glucose? Am J Clin Nutr 1992;55:1033–44.	121
20	Gagnon J, Mauriège P, Roy S, Sjöström D, Chagnon YC, Dionne FT, Oppert JM, Pérusse L, Sjöström L, Bouchard C. The Trp64Arg mutation of the b3 adrenergic receptor gene has no effect on obesity phenotypes in the Québec Family Study and Swedish Obese Subjects cohorts. J Clin Invest 1996;98:2086–93.	116
21	Tremblay A, Simoneau JA, Bouchard C. Impact of exercise intensity on body fatness and skeletal muscle metabolism. Metabolism 1994;43:814–8.	115
22	Chagnon YC, Chen WJ, Pérusse L, Chagnon M, Nadeau A, Wilkison WO, Bouchard C. Linkage and association studies between the melanocortin receptors 4 and 5 genes and obesity-related phenotypes in the Québec Family Study. Mol Med 1997;3:663–73.	108
23	Katzmarzyk P, Pérusse L, Malina RM, Bergeron J, Després JP, Bouchard C. Stability of indicators of the metabolic syndrome from childhood and adolescence to young adulthood: the Quebec Family Study. J Clin Epidemiol 2001;54:190–5.	108
24	Pérusse L, Rice T, Chagnon YC, Després JP, Lemieux S, Roy S, Lacaille M, Ho-Kim MY, Chagnon M, Province MA, Rao DC, Bouchard C. A genome-wide scan for abdominal fat assessed by computed tomography in the Quebec Family Study. Diabetes 2001;50:614–21.	107
25	Pérusse L, Tremblay A, Leblanc C, Cloninger CR, Reich T, Rice J, Bouchard C. Familial resemblance in energy intake: contribution of genetic and environmental factors. Am J Clin Nutr 1988;47:629–35.	104

## Methods

Initial funding for the establishment of QFS was obtained by Claude Bouchard from the Quebec Health Research Council in 1978 and the study was launched at Laval University (Quebec City, Canada) the same year. In phase 1 of the study (1979 to 1982), a total of 1650 individuals from 375 families (nuclear families with biological or adopted offspring and uncle/aunt when available) were recruited and assessed. Recruitment was conducted irrespective of body weight during phase 1, resulting in a cohort with a wide range of body mass index levels. In the course of phase 1, it became evident that the resources of QFS were particularly useful to address questions pertaining to human obesity, especially the genetics of the common form of the disease. This led to a modification of the sampling scheme in order to generate data that would be even more relevant to overweight and obesity. In phase 2 (1989–1997), 385 subjects from 105 phase 1 families were retested, and an additional 74 families ascertained for obesity (at least one parent and one offspring with a body mass index of 32 kg/m^2^ or higher) were added to the cohort. In phase 3 (1998–2002), 44 new families ascertained for obesity were added, while 204 subjects from phase 1 were tested a third time and 113 subjects from phase 2 were tested a second time. Families were all of French descent and were living for the most part within 80 km of Quebec City. Details on recruitment procedures and other aspects of QFS can be found in a previous review [1] and other publications that have appeared since then (see Supplementary material).

## Role of Genetics in the Etiology of Obesity

Obesity is a complex trait resulting from multiple interactions between genetic and behavioral factors. A comprehensive investigation of the genetic determinants of obesity requires appropriate measurements of body composition and fat distribution and information about behavioral factors causing obesity, such as dietary and/or physical activity habits. With its panel of measures of body fatness and fat distribution, extensive phenotyping on the causes and consequences of obesity and with its data on relatives by descent or adoption, the QFS has been one of the early studies that contributed to our understanding of the role of genetic factors in obesity. In this section, we briefly review some of the findings derived from QFS pertaining to the role of genetic factors on body fatness, fat distribution and for phenotypes related to energy intake and energy expenditure.

### Familial Resemblance and Genetic Effects for Body Fatness and Fat Distribution

The first QFS papers pertaining to the genetics of obesity were published in the early 1980s and have shown significant familial resemblance for various phenotypes of obesity. Analysis of variance comparing the between- versus within-family variation showed that there were about two to three times more variation between families than within families for body fatness and body composition as well as for energy intake and energy expenditure, and that familial correlations were generally higher in subjects sharing both genes and environment than in those sharing only the familial environment [2–5]. Given the presence of familial resemblance for obesity-related phenotypes, further studies were undertaken to determine the extent to which this familial resemblance could be attributed to genetic differences and to assess the heritability of the underlying phenotypes. Table 2 presents a summary of familial correlations and heritability estimates for various obesity-related traits based on data from QFS. In that table, the heritability estimate (H^2^) is the multifactorial heritability, analogous to a broad sense heritability as opposed to a narrow sense coefficient, which represents the percentage of variance that is due to all shared familial effects, both genetic and common environment, transmitted from parents to offspring. In some studies where both nuclear and adopted families were used in the analyses, it was possible to distinguish between genetic and common environment and assess a so-called *cultural* heritability, which is the percentage of variance due to the transmission of familial environmental factors.

**Table 2:** Familial correlations and heritability estimates for obesity-related phenotypes in the Quebec Family Study

Variable	Spouses	Parent-offspring	Siblings	H^2a)^	Reference
**Body fat and fat distribution**					
Body mass index	NS	0.23	0.26	40 %	[6]
Sum of 6 skinfolds (SF6)	NS	0.22	0.26	38 %	
Percent body fat	0.20	0.23	0.17	55 %	
Fat mass	0.16	0.22	0.16	48 %	
Fat-free mass	0.21	0.24	0.26	45 %	
TER ^b)^	NS	0.31	0.36	60 %	
Waist circumference (WC)	0.32	0.39	0.26	57 %	Unpublished data
WC adjusted for BMI	0.11	0.26	0.31	51 %	
Total abdominal fat ^c)^	NS	0.26	0.26	52 %	[8]
Subcutaneous abdominal fat	NS	0.21	0.21	42 %	
Visceral abdominal fat	NS	0.28	0.28	56 %	
**Energy intake and eating behaviors**					
Energy intake/kg	0.31	0.26	0.30	30 %	[9]
Carbohydrate (%)	0.50	0.29	0.37	36 %	
Lipid (%)	0.45	0.31	0.36	39 %	
Protein (%)	0.28	0.27	0.38	44 %	
Cognitive dietary restraint	0.17	0.03	0.03	6 %	[10]
Disinhibition	0.09	0.09	0.09	18 %	
Susceptibility to hunger	0.15	0.15	0.15	28 %	
**Energy expenditure and physical activity (PA) level**					
PA level	0.18	0.16	0.42	27 %	[12]
Exercise participation	0.16	0.09	0.34	12 %	
Inactivity	0.13	0.13	0.13	25 %	[13]
Moderate to strenuous PA	0.22	0.16	NS	16 %	
Total daily activity	0.25	0.10	0.10	19 %	
Leisure-time PA (h/week)	0.43	0.09	0.09	17 %	
Resting metabolic rate	0.27	0.24	0.30	47 %	[11]
Respiratory quotient	0.16	0.15	0.16	36 %	

^a^Multifactorial heritability, representing the transmission of both genetic and familial environmental factors.

^b^TER = trunk-to-extremity skinfold ratio [(subscapular + suprailiac + abdominal skinfolds)/(triceps + biceps + medial calf skinfolds)].

^c^Abdominal fat measured by computed tomography at L4-L5 level

The first study published in the literature on heritability estimates for body composition measurements was derived from QFS [6]. This study, based on data from 409 families comprising relatives by descent or adoption, revealed that 45 to 55 % of the variance in percent body fat, fat mass and fat-free mass could be accounted for by the transmission of genetic and familial environmental factors. For body mass index (BMI), subcutaneous fat assessed by the sum of six skinfolds (SF6) and the trunk-to-extremity skinfold ratio (TER), the heritability estimates were 40 %, 38 %, and 60 %, respectively. The variance attributable to genetic factors alone was lower and ranged from about 5 % for SF6 to 30 % for fat-free mass [6]. The familial aggregation of subcutaneous fat patterning was also investigated in QFS by performing a principal component analysis of six skinfolds that led to the identification of two principal components. The heritability of the first component (PC1), indexing total adiposity, reached 46 %, while the heritability of the second component (PC2), contrasting trunk-to-extremity skinfolds, reached 52 % [7]. The heritability of abdominal fat measured by computed tomography was first reported in QFS and results showed significant genetic effects for abdominal fat after adjustment for total body fatness with heritability estimates ranging from 42 % for subcutaneous abdominal fat to 56 % for abdominal visceral fat [8].

In summary, QFS was the first study to investigate the role of familial resemblance and potential genetic effects for several adiposity phenotypes including patterns of subcutaneous fat distribution, and abdominal and visceral adiposity. It was found that there are about two to three times more variation between families than within families for multiple indicators of body fatness with heritability estimates ranging from about 35 to 60 %.

### Familial Resemblance and Genetic Effects for Energy Intake and Energy Expenditure

In order to elucidate the genetic basis of obesity, it is also important to investigate the phenotypes involved in the causal pathways leading to fat deposition. As shown in Table 2, studies based on QFS data have shown significant familial resemblance for phenotypes such as reported energy and macronutrient intake, eating behavior traits, physical activity level and metabolic rates. The familial correlations presented in Table 2 for reported total caloric intake, as well as for the percentage of energy derived from each of the macronutrients, along with the significant spouse, sibling and parent-offspring correlations, confirm that the familial resemblance is the result of both genetic and common environmental effects. Using data from various types of relatives by descent or adoption, we showed heritability estimates in the range of 30 to 44 %, but the additive genetic effect was not significant for energy intake and ranged from 11 to 20 % for macronutrient intake [9]. Data from QFS have also revealed the presence of a significant familial component for three eating behavioral phenotypes (cognitive dietary restraint, disinhibition and susceptibility to hunger) assessed using the Three-Factor Eating Questionnaire, but the heritability estimates were small (6 to 28 %) and mostly accounted for by common familial environment rather than additive genetic effects [10].

Three studies from this cohort reported heritability estimates for phenotypes related to energy expenditure [11–13]. Using a 3-day activity record indexing all daily activities on a scale from 1 (resting energy expenditure) to 9 (high-intensity manual work or exercise) and data on 1610 subjects from 375 families including various types of relatives by descent or adoption, we have shown significant familial resemblance for physical activity level (3-day average sum of scores 1 to 9) and exercise participation (3-day average value of the number of scores 6, 7, 8, or 9) with multifactorial heritability estimates of 27 % and 12 %, respectively [12]. In the case of exercise participation, this heritability was entirely accounted for by common familial environment (cultural heritability = 12 %), while for physical activity level it was mainly accounted for by genetic factors (genetic heritability = 20 %). Familial aggregation of physical activity level was also investigated in another study using only subjects from nuclear families [13]. Three physical activity phenotypes were derived from the 3-day activity record by summing over the three days activity scores: inactivity (sum of scores 1 to 4), moderate to strenuous physical activity (sum of scores 5 to 9) and total daily activity (sum of scores 1 to 9). Past year leisure-time physical activity was also assessed by questionnaire and expressed as the time spent (in hours per week) practicing the most common leisure-time physical activity during the past year. For these physical activity phenotypes, the heritability estimates ranged from 16 % for moderate to strenuous physical activity to 25 % for inactivity, but for all phenotypes, spouses’ correlations were equal or higher than parent-offspring and sibling correlations, suggesting that the heritability of physical activity level likely is strongly influenced by the contribution of common familial environmental factors. We also reported significant heritability estimates of 47 % and 36 % for resting metabolic rate and respiratory quotient, respectively, measured by indirect calorimetry [11].

### Other Genetic Effects Affecting Obesity-related Phenotypes

The heritability of obesity-related phenotypes presented in Table 2 results from to the additive effects of many genes (polygenic effect), each having a small effect on the phenotype, plus other effects such as gene-gene interactions, gene-behavior/environment interactions, major gene effects and others. The hypothesis that there are single genes with a large impact on the phenotype can be modeled in genetic analysis of complex traits. These major gene effects are assumed to result from the segregation at a single gene of two alleles transmitted from parents to offspring according to Mendelian expectations. In the presence of such a major gene effect, the distribution of the phenotype is represented by a mixture of distributions consistent with the segregation of three genotypes instead of the normal distribution associated with a polygenic effect. The first study investigating the contribution of major gene effects for body composition was actually performed in QFS and results showed that fat mass and percent body fat were both influenced by a major gene accounting for 45 % of the variance with multifactorial heritability estimates of 22 % and 26 %, respectively [14]. Major gene effects accounting for as much as 37 % and 51 % of the phenotypic variance, respectively, were also found for the trunk-to-extremity skinfold ratio adjusted for fat mass [15] and for abdominal visceral fat [16]. A study based on QFS data was also the first to investigate the nature of genetic effects affecting resting metabolic rate and to show the presence of a major gene effect accounting for 57 % of the variance in resting metabolic rate after adjustment for fat-free mass and fat mass [17].

In addition to be influenced by a unique set of genes, obesity-related traits can also share common genetic influences. Studies performed in QFS have examined the familial clustering of body fatness and various obesity-related traits such as abdominal fat [18], resting metabolic rate [19], blood pressure [20], fasting glucose and insulin levels [21], and blood lipids [22]. Results have revealed significant evidence of a shared genetic basis between body fat and abdominal fat (bivariate H^2^ of 43 %), resting metabolic rate (bivariate H^2^ of 34 %), diastolic blood pressure (bivariate H^2^ of 33 %), and fasting glucose and insulin levels (bivariate H^2^ of 10 %), while no significant common genetic effects were found between blood lipids and adiposity measures.

### Genes and Molecular Markers of Human Obesity

Association and linkage studies based on candidate genes or molecular markers spanning the whole genome have been major tools used to identify genes influencing the common form of obesity in humans. Since the first study from QFS showing that there was no evidence of association between the A, B and C loci of the HLA system and various body fatness and fat distribution traits [23], a good number of association and linkage studies have been undertaken using data from QFS. It is beyond the scope of this report to review all these findings but such reviews can be found elsewhere [24–26]. Since linkage studies require family data, the primary contribution of our cohort to the identification of genes related to obesity came from genome-wide linkage studies. Genome-wide linkage studies of body fatness [27], fat-free mass [28], abdominal fat assessed by CT scan [29], energy and macronutrient intakes [30], eating behaviors [31], physical activity level [32] and resting metabolic rate and respiratory quotient [11] have been undertaken in QFS. Table 3 presents a summary of the strongest evidence of linkage for these obesity-related traits. The gene most likely responsible for the evidence of linkage (the gene closest to the linkage peak) is also indicated in the table.

**Table 3:** Best evidence of linkage with obesity-related traits derived from genome-wide linkage studies undertaken in the Quebec Family Study

Location	Marker	Trait	Gene	Reference
1q43	D1S184	BMI, FM, % body fat	RGS7	[27]
15q26.2-q26.3	IGF1R CA repeat	FFM	IGF1R	[28]
12q24.3	D12S2078	ASF adjusted for FM	HNF1	[29]
3q27.3	D3S1262	EI, Lipid, CHO	ADIPOQ	[30]
15q24-q25	D15S206	Disinhibition, Hunger	NMB	[31]
2p22-p16	D2S2347	Inactivity	NA	[32]
3q26.1	D3S1763	RMR	GLUT2	[11]
14q22.2	D14S587	RQ	NA	

Traits: FM = fat mass; FFM = fat-free mass; ASF = abdominal subcutaneous fat; EI = energy intake; Lipid = lipid intake; CHO = carbohydrate intake; RMR = resting metabolic rate; RQ = respiratory quotient.

Genes: RGS7 = regulator of G-protein signaling 7; IGFR1 = insulin-like growth factor 1 receptor; ADIPOQ = adiponectin; NMB = neuromedin-B; GLUT2 = Glucose transporter 2.

NA: no gene could be identified

The chromosomal regions identified through genome-wide linkage studies are large and may harbor several genes and fine mapping analyses with tests of association with candidate genes are needed as follow-up. This has been done for two of the chromosomal regions identified in Table 3. In the case of the linkage observed on chromosome 1q43 with body fatness, fine mapping of the region revealed that a polymorphism in the regulator of G-protein signaling 7 (RSG7) gene was associated with body fatness [27]. In our genome-wide linkage study of eating behaviors, the peak linkage found on chromosome 15q24-q25 with disinhibition and susceptibility to hunger was in a region harboring the neuromedin-beta (NMB) gene, a gene encoding a peptide known to inhibit food intake in rats and to modulate behaviors when administered centrally. A missense mutation located in exon 2 of the NMB gene and changing amino acid proline to threonine at position 73 (P73T) was genotyped in all subjects and found to be significantly associated with both eating behavior traits. Moreover, the mutation was found to be associated with 6-year changes in body fatness and gains in fat mass were about three times higher in subjects homozygotes (T73T) for the mutation (about 3 kg) compared to other genotypes (about 1 kg in P73P and P73T subjects) [31]. This study was the first to provide evidence that a gene affecting eating behaviors was associated with a predisposition to obesity.

More recently, a genome-wide association study (GWAS) was performed on the QFS cohort using the Illumina 610-Quad chip containing more than 500,000 single-nucleotide polymorphisms (SNPs) that were tested for association with various obesity-related phenotypes [33]. Due to the availability of GWAS data, QFS is now involved in several international consortia aimed at the identification of obesity-related genes and at confirming or refuting the presence of gene-environment interactions for various obesity-related phenotypes as exemplified in recent studies [34–36].

In summary, we were able to evidence the contribution of a number of candidate genes to human variation in adiposity phenotypes and other obesity-related traits including eating behavior traits. Because of its extensive panel of behavioral and lifestyle measurements, QFS has become an important resource in the search for gene-behavior interaction effects on obesity-related traits.

## Behavioral and Environmental Determinants of Obesity

### Commonly Recognized Correlates of Obesity

An objective of QFS was to examine the association between lifestyle and cardiometabolic health. One of the early decisions that had to be taken was related to the selection of the tools to be used in assessing dietary and activity behavior.

Despite its inherent limitations, the 3-day dietary record method was selected to document usual nutrient intake. It was perceived as a good compromise between the burden imposed to the participants and the ability to obtain information on food habits under free-living conditions. Its reproducibility was studied in 61 individuals who completed the dietary records twice separated by an interval of 7 days [37]. The reliability of these dietary records was found to be moderate to high for most nutrients. As further discussed herein, the 3-day dietary record based on two weekdays and one weekend day has been very useful in documenting behavioral correlates of obesity in QFS.

The study of physical activity and its related energy cost required the development of a new tool, a 3-day physical activity diary, which was administered over the same days as those during which the food diary was completed. As mentioned earlier, physical activities were classified on a 1 to 9 scale on the basis of their estimated energy cost [38]. The reliability of this diary was tested under the same conditions as the food diary and was found to be very high, as reflected by an intra-class correlation coefficient of 0.96 for estimated mean energy expenditure over 3 days. The analysis of the relationship between energy expenditure, physical work capacity, and body fatness provided additional support for the validity of this approach.

Data of this cohort were used extensively to examine the relationship between body fatness and habitual dietary fat intake. These analyses showed a significant positive association between fat mass or subcutaneous adiposity and percent energy intake as lipid. When comparing the upper and lower quartiles of percent energy as lipid, a significantly greater energy intake was observed in the upper quartile, which also exhibited a mean fat mass level exceeding by 5 kg the value observed in subjects of the lowest quartile [39]. Further analyses showed that habitual alcohol intake was also related to an increased energy intake. In accordance with experimental data, alcohol intake had no inhibitory effect on lipid intake whereas reduced carbohydrate consumption was observed in high alcohol consumers [40].

The data of the QFS cohort were also analyzed to explore the impact of a healthy lifestyle on body fat. For that purpose, low and high lipid consumers were compared and their comparison was repeated while also considering alcohol consumption and vigorous physical activity participation [41]. The results showed that when individuals reporting low fat and low alcohol intake as well as regular vigorous physical activity participation were compared to those displaying opposite behaviors, the between-group difference in subcutaneous adiposity was doubled compared to when subjects were only compared on the basis of lipid intake. Interestingly, this increase in the between-group difference in subcutaneous fat was essentially explained by an increase in truncal subcutaneous fat.

Recent analyses of the data from QFS also allowed establishing some links with relevant biomarkers. The use of an oral glucose tolerance test at baseline revealed that insulin concentration at 30 minutes post oral glucose ingestion, as a proxy measure of insulin secretion, predicted weight gain and change in waist circumference over 6 years in low fat consumers but to a lesser extent in the highest dietary fat intake group [42].

The physical activity diary also allowed for the study of the effects of physical activity intensity on body fat. Thus, in a subsample of 352 healthy men, percent body fat and subcutaneous adiposity were significantly lower in those reporting vigorous physical activity than in those not performing such activities. A separate study suggested that this potential effect of exercise intensity might be partly explained by an increase in post-exercise resting metabolic rate [43].

In addition to documenting common correlates of obesity, QFS had a substantial impact in the investigation of indicators of adipose tissue distribution. For instance, Després et al. [44] reported that measurement of upper body adiposity should be considered when interpreting the blood lipid profile, especially in males. Lemieux et al. [45] observed that the greater health hazards associated with excess adiposity in men than in women may be explained by the fact that premenopausal women can accumulate more body fat than men of comparable age before reaching the amounts of visceral adipose tissue found in men. In a subsequent study, Pouliot et al. [46] reported that waist circumference and abdominal sagittal diameter could be used as indicators of abdominal visceral adipose tissue accumulation and related cardiovascular risk. This paper is still the most frequently cited contribution of QFS with over 950 citations (see Table 1). We subsequently demonstrated that waist circumference is a more convenient anthropometric correlate of visceral adipose tissue than waist-to-hip ratio because threshold values of waist girth corresponding to critical amounts of visceral adipose tissue do not appear to be influenced by sex or by the degree of obesity [47]. These findings are consistent with another QFS study showing that waist and hip circumferences have independent and opposite effects on cardiovascular disease risk factors [48].

In summary, QFS contributed multiple new findings on the associations of dietary lipid intake, alcohol intake, and exercise intensity with adiposity and the risk of obesity. The relationships between abdominal adiposity and visceral adipose tissue with cardiovascular and diabetes risk factors were extensively documented in QFS.

### Emerging Correlates of Obesity

The positive energy balance underlying obesity is generally attributed to excess energy intake and low levels of physical activity related energy expenditure. Unhealthy diet and physical inactivity are thus the two major factors upon which preventive and therapeutic programs for obesity are focused. The influence of these “traditional” risk factors for obesity has been largely documented, including in QFS. However, recent research has emphasized that other, less obvious factors such as short sleep duration and low micronutrient intake may also associate with obesity-related traits [49–51]. The identification of all putative contributors to the obesity epidemic is critical to our understanding of the conditions under which weight gain occurs.

QFS projects have shown that low calcium [52] and micronutrient [53] intakes, high dietary restraint behavior [54], high disinhibition and susceptibility to hunger behaviors [55], and short sleep duration [56, 57] are all associated with excess adiposity and/or obesity. Interestingly, these risk factors for obesity do not have any caloric value *per se* but appear to promote a positive energy balance by mechanisms that remain to be established.

**Fig. 1:**
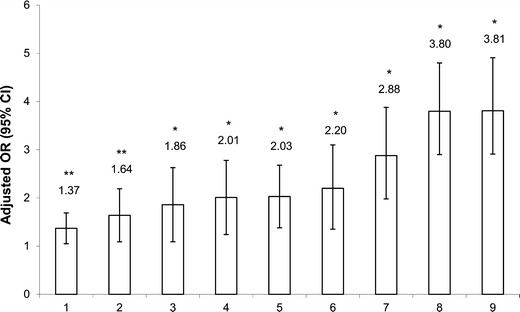
Associations between risk factors and adult overweight/obesity in the cross-sectional sample. Logistic regression was used and ORs were determined for the “at risk” compared to the “reference” groups of risk factors for the odds of having a body mass index greater than 25 kg/m^2^. Model adjusted for age, sex, and socioeconomic status. Legend of the *x* axis: 1 = high alcohol intake (≥10 g/day vs. 0 g/day), 2 = high dietary lipid intake (≥40 % fat/day vs. <30 % fat/day), 3 = non-consumption of multivitamin and dietary supplements (vs. consumer), 4 = high dietary restraint behavior (≥8 restraint score vs. ≤4 restraint score), 5 = non-participation in high-intensity physical activity (vs. ≥30 min/day), 6 = high susceptibility to hunger behavior (≥5 hunger score vs. ≤2 hunger score), 7 = low dietary calcium intake (<600 mg/day vs. ≥1,000 mg/day), 8 = high disinhibition eating behavior (≥6 disinhibition score vs. ≤3 disinhibition score) and 9 = short sleep duration (<6 hours/day vs. 7–8 h/day). OR, odds ratio; CI, confidence interval. *n* = 537 (230 men and 307 women). *P < 0.01; **P < 0.05. Figure adapted from Chaput et al. [58]

**Fig. 2:**
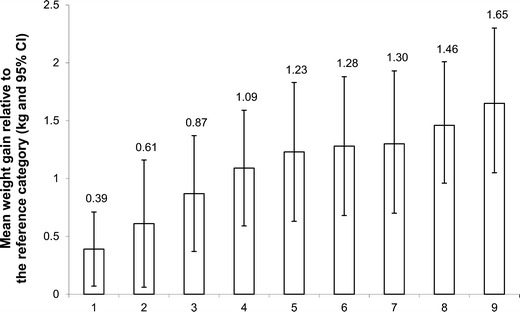
Mean weight gain above baseline weight over the 6-year follow-up period for individuals with the risk factor relative to the reference category. Model adjusted for age, sex, baseline body mass index, length of follow-up, socioeconomic status, and all other risk factors. Legend of the *x* axis: 1 = high alcohol intake (≥10 g/day vs. 0 g/day), 2 = high dietary lipid intake (≥40 % fat/day vs. <30 % fat/day), 3 = non-consumption of multivitamin and dietary supplements (vs. consumer), 4 = high dietary restraint behavior (≥8 restraint score vs. ≤4 restraint score), 5 = non-participation in high-intensity physical activity (vs. ≥30 min/day), 6 = high susceptibility to hunger behavior (≥5 hunger score vs. ≤2 hunger score), 7 = low dietary calcium intake (<600 mg/day vs. ≥1,000 mg/day), 8 = high disinhibition eating behavior (≥6 disinhibition score vs. ≤3 disinhibition score) and 9 = short sleep duration (<6 hours/day vs. 7–8 h/day). CI, confidence interval. *n* = 283. Figure adapted from Chaput et al. [58]

We have examined the independent associations of these risk factors with overweight/obesity using cross-sectional (*n* = 537) and longitudinal (*n* = 283, 6-year follow-up period) samples of the QFS adult participants, aged 18–64 years [58]. As shown in Fig. 1, short sleep duration (self-reported), high disinhibition eating behavior (Three-Factor Eating Questionnaire), and low dietary calcium intake (3-day dietary record) were more strongly associated with adult overweight and obesity than commonly recognized risk factors as represented by high dietary lipid intake (3-day dietary record) and non-participation in high-intensity physical activity (3-day physical activity record). After adjustment for age, socioeconomic status, and all other risk factors as covariates, only short sleep duration, high disinhibition eating behavior, and low dietary calcium intake were significantly and independently associated with overweight and obesity in both sexes. A similar pattern was observed for weight gain over a 6-year follow-up period. Short-duration sleepers, low calcium consumers, and those with a high disinhibition eating behavior trait were more likely to gain weight over the 6-year follow-up period (Fig. 2).

Subsequently, we compared the relative risks of two traditional (high dietary lipid intake and non-participation in high-intensity physical activity) versus three emerging (short sleep duration, high disinhibition eating behavior, and low dietary calcium intake) risk factors for overweight and obesity development over 6 years of follow-up and tested whether combinations of these risk factors yielded higher relative risk levels [59]. As shown in Table 4, the risk of developing overweight and obesity tended to be higher for the emerging risk factors compared to the indicators of diet and physical activity level. However, the confidence intervals of the ORs for the common and emerging risk factors overlap in all direct pairwise comparisons. Although the results of this study are suggestive of substantial contributions to the risk of excessive weight gain by both types of risk factors, the lack of statistical power prevents us from concluding on the relative importance of common versus emerging risk factors at this time.

**Table 4:** Traditional versus nontraditional risk factors and incidence of adult overweight and obesity in the Quebec Family Study

	OR	95 % CI
***Common risk factors***		
High lipid intake		
≥40% fat/day (vs. <30 % fat/day)	1.31	0.81–1.82
Nonparticipation in high-intensity physical activity		
0 min/day (vs. ≥30 min/day)	1.80**	1.18–2.47
Two risk factors combined		
≥40% fat/day + 0 min/day	2.66*	1.59–3.79
***Emerging risk factors***		
Low calcium intake		
<600 mg/day (vs. ≥1,000 mg/day)	2.18*	1.17–3.26
High disinhibition eating behavior		
≥6 disinhibition score (vs. ≤3 disinhibition score)	2.76*	1.48–4.10
Short sleep duration		
<6 h/day (vs. 7–8 h/day)	2.97*	1.68–4.34
Low calcium intake and high disinhibition eating behavior		
<600 mg/day + ≥6 disinhibition score	3.76*	2.31–5.39
Low calcium intake and short sleep duration		
<600 mg/day + <6 h/day	4.02*	2.71–5.46
High disinhibition eating behavior and short sleep duration		
≥6 disinhibition score + <6 h/day	4.49*	3.06–6.06
Three risk factors combined		
<600 mg of calcium/day + ≥6 disinhibition score +		
<6 h of sleep/day	4.92*	3.22–6.73

Odds ratios (OR) and confidence intervals (CI) calculated by logistic regression analysis. Model adjusted for age, sex, baseline body mass index, length of follow-up, and socioeconomic status. ORs were determined for the “at risk” compared to the “reference” groups of risk factors for the odds of developing overweight/obesity (i.e. BMI ≥ 25 kg/m^2^) over the 6-year follow-up period among the participants who were not overweight or obese at baseline. *P < 0.01; **P < 0.05. (Table adapted from Chaput et al. [59])

The emerging risk factors identified in QFS have received increased attention in recent scientific literature. For instance, there is accumulating evidence supporting the role of reduced sleep as a contributor to obesity in adults and children [60]. The mechanisms by which short sleep duration may predispose to weight gain are still under investigation but could involve both sides of the energy balance equation. Experimental sleep restriction has been reported to increase appetite via an up-regulation of appetite-stimulating hormones [61]. Lack of sleep could also lead to weight gain and obesity by increasing the time available for eating and by making the maintenance of a healthy, physically active lifestyle more difficult [62]. In an environment where energy-dense foods are highly palatable and readily available, caloric intake may be directly proportional to the time spent awake, especially if most of wakefulness is spent in screen-based sedentary activities where snacking is common [63]. Furthermore, the increased fatigue and tiredness associated with not having enough sleep may impact overall physical activity participation [64].

We recently showed, using the 6-year longitudinal design of QFS, that short sleep duration preferentially increases abdominal adiposity rather than overall adiposity [65]. Because stress-induced hypothalamo-pituitary-adrenal (HPA) axis activation has been shown to play a role in body fat accumulation in the abdominal region [66, 67], the HAP axis hyperactivity associated with short sleep duration could be an important mechanism involved. If insufficient sleep predominantly activates the HPA axis over the sympathetic nervous system, abdominal fat accumulation may become a normal consequence of short sleep duration. Interestingly, preliminary findings from QFS lend support to the concept that increasing sleeping time in short-duration sleepers has the potential to limit adiposity gain over time [68]. Specifically, we observed that a spontaneous change in sleep duration from a short (≤6 h per day) to a healthier length (7–8 h per day) was associated with an attenuation of fat mass gain over the 6-year follow-up period. In summary, the preponderance of the evidence to date suggests that having a good night’s sleep should be encouraged as an adjunct measure in the prevention of obesity.

Another emerging risk factor for excess body weight gain and overweight/obesity incidence is disinhibited eating. Disinhibition as an eating behavior trait reflects a tendency toward overeating and eating opportunistically and is assessed in QFS using the Three-Factor Eating Questionnaire developed by Stunkard and Messick [69]. Examples include eating in response to negative affect, overeating when others are eating, not being able to resist stimulation to eating and overeating in response to the palatability of the food. Disinhibited eating behavior is not only associated with weight gain and obesity, but also with mediating variables such as less healthful food choices, which contribute to a state of positive caloric balance and poorer health [70–72]. Disinhibited eating is predictive of poorer success at weight loss, and of weight regain after weight loss [73, 74], and is also associated with sedentary behavior [75] and poor psychological health [76]. Thus, disinhibited eating is important to consider as it may significantly impact energy balance and lead to obesity development.

Another emerging correlate of body weight gain in QFS is low dietary calcium intake. Others have reported an effect of dietary calcium on energy and fat balance [77, 78]. We reported earlier based on data from the QFS [52] that a low daily calcium intake was associated with greater adiposity, particularly in women. It has been proposed that dietary calcium binds lipids in the gastrointestinal tract and keeps them unavailable for absorption, which in turn reduces post-prandial lipemia and increases fecal fat excretion [79, 80]. Chronic high calcium intake has also been shown to increase fat oxidation in adults [81]. Another potential mechanism could be related to appetite control. It has been shown in animal models that calcium deficiency results in “calcium-seeking” behavior, which may result in increased food intake [82]. Humans possess taste receptors for calcium in the gastrointestinal tract and signaling from these receptors may be linked to appetite control [83]. Recent human studies have indeed provided support for a modest effect of calcium intake on appetite control [84–86]. Although the evidence is suggestive, we need to better understand the role of dietary calcium on energy balance before recommending its use in the prevention or treatment of obesity.

The findings from QFS summarized herein are suggestive of the importance of emerging factors in the etiology of obesity. The findings also support the notion that caloric intake and physical activity behavior, two commonly recognized risk factors for excessive weight gain and obesity, are also contributors to the risk profile as one would expect. The key limitations of QFS need to be highlighted in order to put these observations into perspective. First, the study is observational and the results could have been affected by undefined confounders. As is well known, cause and effect relationships cannot be established from observational data. Second, we need to keep in mind that the risk factors for obesity discussed in this review are self-reported and not directly measured, except those that were assayed in plasma samples. There are obvious limitations associated with self-reported measures (e.g., recall and social desirability bias). Finally, the external generalizability of our findings may be restricted to adults of European descent.

## Conclusion

In summary, QFS has contributed significantly over the last three decades to our understanding of the etiology of obesity. The study provided some of the important early data on the potential role of genetic factors in the predisposition to the common forms of obesity including abdominal and visceral adiposity, well before it became a topic of general interest. Significant familial aggregation and heritability level were observed not only for BMI, total adiposity, fat-free mass, subcutaneous fat distribution, abdominal fat and visceral adipose tissue level but also for metabolic rates, physical activity level, macronutrient intake and eating behavior traits. Several DNA markers were found to be associated with these phenotypes and evidence of gene-behavior interaction effects was reported. QFS also contributed substantially to our understanding of the importance of obesogenic behavior to the risk of obesity, including the demonstration that dietary lipid intake, alcohol consumption and exercise intensity significantly influence adiposity. In recent years, we reported evidence that nontraditional risk factors for obesity, especially short sleep duration, should receive more attention. Collectively, we believe that QFS has contributed in a significant manner to the effort of the scientific community aimed at reaching a better understanding of the biology and behaviors predisposing to obesity and has offered some testable leads on how to address the complex human obesity problem.

## Electronic supplementary material

Below is the link to the electronic supplementary material.

ESM 1 (DOCX 53 kb)
