# Supplementary material for: Findings from the Quebec Family Study on the Etiology of Obesity: Genetics and Environmental Highlights
Source: Curr Obes Rep. 2014 Jan 4;3(1):54–66. doi: 10.1007/s13679-013-0086-3 (PMC3920031; doi:10.1007/s13679-013-0086-3)
Supplement: Supplementary file 1 — (DOCX 53 kb) [file 13679_2013_86_MOESM1_ESM.docx]

**Papers published since 1981 based in full or
in part on the data from the Quebec Family Study**

**Total papers = 276, Total citations = 12,001
as of Sept. 10, 2013**

|  |
| --- |
| 1. Chagnon, Y, Bouchard C, Allard C. Isoelectric focusing of red cell phosphoglucomutase (E.C.: 2.7.5.l) at the PGM1 locus in a French-Canadian population. Hum Genet 59: 36-8, 1981. |
| 1. Bouchard C. Family resemblance in selected biological traits: a preliminary report. In: Lavallée H, Shephard RJ, editors. Croissance et développement de l'enfant Child growth and development. Trois Rivières: Université du Québec à Trois-Rivières; 1982. p. 122-30. |
| 1. Lortie G, Bouchard C, Leblanc C, Tremblay A, Simoneau JA, Thériault G, and Savoie JP. Familial similarity in aerobic power. Hum Biol 54: 801-12, 1982. |
| 1. Allard C, Leblanc C, Talbot J, Leclerc S, Bouchard C. Familial aggregation of PWC150, blood lipid composition and serum uric acid in adopted and biological siblings. In: Knuttgen HG, Vogel JA, Poortmans J, editors. Biochemistry of exercise. International series on sport sciences, vol. 13. Champaign, IL: Human Kinetics Publishers; 1983. p. 219-24. |
| 1. Bouchard C, Leblanc J, Côté J, Jobin J, Jobin M, Labrie A, Leblanc C. Familial resemblance in catecholamine changes to cold stress and maximal exercise. Hum Hered, 33: 170-8, 1983. |
| 1. Bouchard C, Tremblay A, Leblanc C, Lortie G, Savard R, Thériault G. A method to assess energy expenditure in children and adults. Am J Clin Nutr 37: 461-7, 1983. |
| 1. Leclerc S, Bouchard C, Talbot J, Gauvin R, Allard C. Association between serum high-density lipoprotein cholesterol and body composition in adult men. Int J Obes 7:555-61, 1983. |
| 1. Lortie G, Bouchard C, Simoneau JA, Thériault G. La consommation maximale d'oxygène: les relations avec l'âge, le sexe, la graisse corporelle et le style de vie. In: Landry F, editor. Health risk estimation, risk reduction and health promotion. Proceedings of the 18th annual meeting of the Society of Prospective Medicine. Ottawa: Canadian Public Health Association; 1983. p. 79-86. |
| 1. Perusse L, Bouchard C, Leblanc C, Thériault G, Tremblay A. Physical Fitness and Lifestyle. In: Landry F, editor. Health risk estimation, risk reduction and health promotion. Proceedings of the 18th annual meeting of the Society of Prospective Medicine. Ottawa: Canadian Public Health Association; 1983. p. 71-8. |
| 1. Savard R, Bouchard C Leblanc C, Tremblay A. Familial resemblance in fatness indicators. Ann Hum Biol 10: 111-8, 1983. |
| 1. Savard R, Bouchard C, Tremblay A, Leblanc C. Familial resemblance for energy intake and energy expenditure and their relationships with body fatness. In: Landry F, editor. Health risk estimation, risk reduction and health promotion. Proceedings of the 18th annual meeting of the Society of Prospective Medicine. Ottawa: Canadian Public Health Association; 1983. p. 50-7. |
| 1. Tremblay A, Leblanc C, Sévigny J, Savoie JP, Bouchard C. The relationship between energy intake and expenditure: a sex difference. In: Landry F, editor. Health risk estimation, risk reduction and health promotion. Proceedings of the 18th annual meeting of the Society of Prospective Medicine. Ottawa: Canadian Public Health Association; 1983. p. 115-9. |
| 1. Tremblay A, Sévigny J, Leblanc C, Bouchard C. The reproducibility of a three-day dietary record. Nutr Res 3: 819-30, 1983. |
| 1. Bouchard C, Lortie G, Simoneau JA, Leblanc C, Thériault G, Tremblay A. Submaximal power output in adopted and biological siblings. Ann Hum Biol 11: 303-9, 1984. |
| 1. Perusse L, Bouchard C, Leblanc C, Tremblay A. Energy intake and physical fitness in children and adults of both sexes. Nutr Res 4:363-70, 1984. |
| 1. Bouchard C. Inheritance of fat distribution and adipose tissue metabolism. In: Vague J, Björntorp P, Guy-Grand B, Rebuffé-Scrive M, Vague P, editors. Metabolic complications of human obesities. Amsterdam: Elsevier Science Publishers B.V; 1985. p. 87-96. |
| 1. Bouchard C. Reproducibility of body composition and adipose- tissue measurements in humans. In: Roche AF, editor. Body-composition assessments in youth and adults. Report of the sixth Ross conference on medical research. Columbus, OH: Ross Laboratories; 1985. p. 9-14. |
| 1. Bouchard C, Perusse L, Rivest J, Roy R, Morissette J, Allard C, Thériault G, Leblanc C, Tremblay A. HLA system, body fat and fat distribution in children and adults. Int J Obes 9:411-22, 1985. |
| 1. Bouchard C, Savard R, Després J-P, Tremblay A, Leblanc C. Body composition in adopted and biological sibs. Hum Biol 57:61-75, 1985. |
| 1. Després J-P, Allard C, Tremblay A, Talbot J, Bouchard C. Evidence for a regional component of body fatness in the association with serum lipids in men and women. Metabolism 34:967-73, 1985. |
| 1. Himes JH, Bouchard C. Do the new Metropolitan Life Insurance weight-height tables correctly assess body frame and body fat relationships?  Am J Public Health 75(9): 1076-9, 1985. |
| 1. Leclerc S, Allard C, Talbot J, Gauvin R, Bouchard C. High density lipoprotein cholesterol, habitual physical activity and physical fitness. Atherosclerosis 57:43-51, 1985. |
| 1. Seoane NA, Roberge AG, Pagé M, Allard C, Bouchard C. Selected indices of iron status in adolescents. J Can Dietetic Assoc 46:298-303, 1985. |
| 1. Couture L, Chagnon M, Allard C, Bouchard C. Esterase D polymorphism in a French-Canadian population. Hum Genet 73:276, 1986. |
| 1. Simoneau JA, Lortie G, Boulay MR, Marcotte M, Thibault MC, Bouchard C. Inheritance of human skeletal muscle and anaerobic capacity adaptation to high-intensity intermittent training. Int J Sports Med 7:167-71, 1986. |
| 1. Bouchard C. Genetics of body fat, energy expenditure and adipose tissue metabolism. In: Berry EM, Blondheim SH, Eliahou HE,Shafrir E, editors. Recent advances in Obes Res: V. Proceedings of the 5th International Congress on Obesity. Jerusalem (Israël). London: John Libbey; 1987. p. 16-25. |
| 1. Bouchard C, Tremblay A. Genetics of body composition and fat distribution. In: Norgan NG, editor. Human Body Composition and Fat Distribution. Report of an EC Workshop, London 10-12 December 1985. Euro Nutr 8:175-88, 1987. |
| 1. Couture L, Chagnon M, Allard C, Bouchard C. Adenosine deaminase, adenylate kinase and acid phosphatase polymorphism in a French-Canadian population. Hum Genet 75:188, 1987. |
| 1. Perusse L, Leblanc C, Tremblay A, Allard C, Thériault G, Landry F, Talbot J, Bouchard C. Familial aggregation in physical fitness, coronary heart disease risk factors, and pulmonary function measurements. Prev Med 16:607-15, 1987. |
| 1. Perusse L, Lortie G, Leblanc C, Tremblay A, Thériault G, Bouchard C. Genetic and environmental sources of variation in physical fitness. Ann Hum Biol 14:425-34, 1987. |
| 1. Bouchard C. Human variation in anthropometric dimensions. In: Lohman TG, Roche AF, Martorell R, editors. Anthropometric Standardization Reference Manual. Champaign, IL: Human Kinetics; 1988. p. 103-5. |
| 1. Bouchard C. Inheritance of human fat distribution. In: Bouchard C, Johnston FE, editors. Fat distribution during growth and later health outcomes. Current Topics in Nutrition and Disease. Vol. 17. New York: Alan R. Liss, Inc; 1988. p. 103-25. |
| 1. Bouchard C. Genetic factors in the regulation of adipose tissue distribution. Acta Med Scand, Suppl. 723:135-41, 1988. |
| 1. Bouchard C, Perusse L, Leblanc C, Tremblay A, Thériault G. Inheritance of the amount and distribution of human body fat. Int J Obes 12:205-15, 1988. |
| 1. Després J-P, Tremblay A, Perusse L, Leblanc C, Bouchard C. Abdominal adipose tissue and serum HDL-cholesterol: association independent from obesity and serum triglyceride concentration. Int J Obes 12:1-13, 1988. |
| 1. Després J-P, Tremblay A, Leblanc C, Bouchard C. Effect of the amount of body fat on the age-associated increase in serum cholesterol. Prev Med 17:423-31, 1988. |
| 1. Després J-P, Tremblay A, Thériault G, Perusse L, Leblanc C, Bouchard C. Relationships between body fatness, adipose tissue distribution and blood pressure in men and women. J Clin Epidemiol 41:889-97, 1988. |
| 1. Malina R, Bouchard C. Subcutaneous fat distribution during growth. In: Bouchard C, Johnston FE, editors. Fat distribution during growth and later health outcomes. Current Topics in Nutrition and Disease. Vol. 17. New York: Alan R. Liss, Inc; 1988. p. 63-84. |
| 1. Perusse L, Tremblay A, Leblanc C, Cloninger CR, Reich T, Rice J, Bouchard C. Familial resemblance in energy intake: contribution of genetic and environmental factors. Am J Clin Nutr 47:629-35, 1988. |
| 1. Bouchard C, Tremblay A, Perusse L, Leblanc C, Thériault G, George V. Cohabitation, activity level and energy intake in parent-child resemblance for selected biological traits. Am J Hum Biol 1:209-15, 1989. |
| 1. Brun LD, Gagné C, Julien P, Tremblay A, Moorjani A, Bouchard C, Lupien PJ. Familial lipoprotein lipase activity deficiency: study of total body fatness and subcutaneous fat distribution. Metabolism 38:1005-9, 1989. |
| 1. George V, Tremblay A, Després J-P, Leblanc C, Perusse L, Bouchard C. Evidence for the existence of small and large eaters of similar fat-free mass and activity level. Int J Obes 13:43-53, 1989. |
| 1. Himes JH, Bouchard C. Validity of anthropometry in classifying youths as obese. Int J Obes 13:183-93, 1989. |
| 1. Perusse L, Després J-P, Tremblay A, Leblanc C, Talbot J, Allard C, Bouchard C. Genetic and environmental determinants of serum lipids and lipoproteins in French Canadian families. Arteriosclerosis 9:308-18, 1989. |
| 1. Perusse L, Rice T, Bouchard C, Vogler GP, Rao DC. Cardiovascular risk factors in a French Canadian population: resolution of genetic and familial environmental effects on blood pressure using extensive information on environmental correlates. Am J Hum Genet 45:240-51, 1989. |
| 1. Perusse L, Tremblay A, Leblanc C, Bouchard C. Genetic and environmental influences on level of habitual physical activity and exercise participation. Am J Epidemiol 129:1012-22, 1989. |
| 1. Rice T, Vogler GP, Perusse L, Bouchard C, Rao DC. Cardiovascular risk factors in a French Canadian population: resolution of genetic and familial environ-mental effects on blood pressure using twins, adoptees, and extensive information on environmental correlates. Genet Epidemiol 6:571-88, 1989. |
| 1. Tremblay A, Plourde G, Després J-P, Bouchard C. Impact of dietary fat content and fat oxidation on energy intake in humans. Am J Clin Nutr 49:799-805, 1989. |
| 1. Rice T, Bouchard C, Borecki IB, Rao DC. Commingling and segregation analysis of blood pressure in a French Canadian population. Am J Hum Genet 46:37-44, 1990. |
| 1. Bouchard C. Genetic aspects of anthropometric dimensions relevant to assessment of nutritional status. In: Himes J, editor. Anthropometric assessment of nutritional status. New York: Alan R. Liss; 1991. p. 213-31. |
| 1. Bouchard C. Editorial. L'obésité est-elle héréditaire? La Revue du Praticien 19:1773-6, 1990. |
| 1. Meyer F, Moisan J, Marcoux D, Bouchard C. Dietary and physical determinants of Menarche. Epidemiology 1:377-81, 1990. |
| 1. George V, Tremblay A, Després J-P, Leblanc C, Bouchard C. Effect of dietary fat content on total and regional adiposity in men and women. Int J Obes 14:1085-94, 1990. |
| 1. Després JP, Tremblay A, Prud’homme D, Bouchard C.  Effets métaboliques de l'évolution de la distribution régionale du tissu adipeux avec l'âge.  Age et nutrition 2:7-18, 1991. |
| 1. Himes JH, Bouchard C, Pheley AM.  Lack of correspondence among measures identifying the Obese.  Am J Prev Med  7:107-11, 1991. |
| 1. Borecki IB, Rice T, Bouchard C, Rao DC.  Commingling analysis of generalized body mass and composition measures:  the Québec Family Study.  Int J Obes 15:763-73, 1991. |
| 1. Mauriège P, Després J-P, Prud’homme D, Pouliot MC, Marcotte M, Tremblay A, Bouchard C. Regional variation in adipose tissue lipolysis in lean and obese men. J Lipid Res 32:1625-33, 1991. |
| 1. Bouchard C.  Genetic aspects of human obesity.  In: Belfiore F, Jeanrenaud B, Papalia D, editors. Obesity: Basic Concepts and Clinical Aspects, Frontiers in Diabetes, Vol 11  Basel: Karger; 1992. p. 28-36. |
| 1. Bouchard C, Tremblay A, Després J-P, Dériaz O, Dionne FT.  The genetics of Body energy content and energy balance:  an overview.  In:  Bray GA and Ryan DH, editors.  The Science of Food Regulation:  food intake, taste, nutrient partitioning and energy expenditure.  Baton Rouge, LA: Louisiana State University Press; 1992. p. 3-21. |
| 1. Bouchard C. Heredity and regional fat distribution during growth. In: Hernandez M, Argente J, editors. Human growth: Basic and clinical aspects. The Netherlands: Elsevier Science BV; 1992. p. 227-32. |
| 1. Després J-P, Moorjani S, Lupien PJ, Tremblay A, Nadeau A, Bouchard C. Genetic aspects of susceptibility to obesity and related dyslipidemias. Molec Cell Biochem 113:151-69, 1992. |
| 1. Spiegelman D, Israel RG, Bouchard C, Willett WC. Absolute fat mass, percent body fat, and body-fat distribution: which is the real determinant of blood pressure and serum glucose? Am J Clin Nutr 55:1033-44, 1992. |
| 1. Lemieux S, Després J-P, Nadeau A, Prud’homme D, Tremblay A, Bouchard C. Heterogeneous glycaemic and insulinaemic responses to oral glucose in non-diabetic men: interactions between duration of obesity, body fat distribution and family history of diabetes mellitus. Diabetologia 35:653-9, 1992. |
| 1. Rice T, Borecki IB, Bouchard C, Rao DC. Commingling analysis of regional fat distribution measures: the Québec family study. Int J Obes 16:831-44, 1992. |
| 1. Rice T, Borecki IB, Bouchard C, Rao DC. Segregation analysis of fat mass and other body composition measures derived from underwater weighing. Am J Hum Genet 52:967-73, 1993. |
| 1. Song TMK, Malina RM, Bouchard C. Familial resemblance in somatotype. Am J Hum Biol 5:265-72, 1993. |
| 1. Rice T, Borecki IB, Bouchard C, Rao DC. Segregation analysis of body mass index in an unselected French-Canadian sample: the Québec Family Study. Obes Res 1:288-94, 1993. |
| 1. Borecki IB, Bonney GE, Rice T, Bouchard C, Rao DC. Influence of genotype-dependent effects of covariates on the outcome of segregation analysis of the body mass index. Am J Hum Genet 53:676-87, 1993. |
| 1. Lemieux S, Prud’homme D, Bouchard C, Tremblay A, Després J-P . Sex differences in the relation of visceral adipose tissue accumulation to total body fatness. Am J Clin Nutr 58:463-7, 1993. |
| 1. Després J-P, Verdon MF, Moorjani S, Pouliot MC, Nadeau A, Bouchard C, Tremblay A, Lupien PJ. Apolipoprotein-E polymorphism modifies relation of hyperinsulinemia to hypertriglyceridemia. Diabetes 42 (10):1474-81, 1993. |
| 1. Vohl MC, Dionne F, Dériaz O, Chagnon M, Bouchard C. Detection of a MspI restriction fragment length polymorphism for the human sex hormone-binding globulin (SHBG) gene. Hum Genet 93:84, 1994. |
| 1. Dériaz O, Dionne FT, Perusse L, Tremblay A, Vohl MC, Côté G, Bouchard C. DNA variation in the genes of the Na,K-Adenosine Triphosphatase and its relation with resting metabolic rate, respiratory quotient, and body fat. J Clin Invest 93:838-43, 1994. |
| 1. Bouchard C, Perusse L. Genetics of obesity: Family studies. In: Bouchard C, editor. The Genetics of Obesity. Boca Raton, FL: CRC Press; 1994. p. 79-92. |
| 1. Borecki IB, Province MA, Bouchard C, Rao DC. Genetics of obesity: Etiologic heterogeneity and temporal trends. In: Bouchard C, editor. The Genetics of Obesity. Boca Raton, FL: CRC Press; 1994. p. 109-23. |
| 1. Perusse L, Bouchard C. Genetics of energy intake and food preferences. In: Bouchard C, editor. The Genetics of Obesity Boca Raton, FL: CRC Press; 1994. p. 125-34. |
| 1. Bouchard C, Dériaz O, Perusse L, Tremblay A. Genetics of energy expenditure in humans. In: Bouchard C, editor. The Genetics of Obesity. Boca Raton, FL: CRC Press; 1994. p. 135-45. |
| 1. Song TMK, Perusse L, Malina RM, Bouchard C. Twin resemblance in somatotype and comparisons with other twin studies. Hum Biol 66:453-64, 1994. |
| 1. Borecki IC, Rice T, Perusse L, Bouchard C, Rao DC. An exploratory investigation of genetic linkage with body composition and fatness phenotypes: The Québec Family Study. Obes Res 2:213-9, 1994. |
| 1. Shephard RJ, Bouchard C. Population evaluations of health related fitness from perceptions of physical activity and fitness. Can J Appl Physiol 19:151-73, 1994. |
| 1. Shephard RJ, Bouchard C. Principal components of fitness: Relationship to physical activity and lifestyle. Can J Appl Physiol 19:200-14, 1994. |
| 1. Tremblay A, Simoneau JA, Bouchard C. Impact of exercise intensity on body fatness and skeletal muscle metabolism. Metabolism 43:814-8, 1994. |
| 1. Pouliot MC, Després J-P, Dionne FT, Vohl MC, Moorjani S, Prud’homme D, Bouchard C, Lupien PJ. ApoB-100 gene EcoRI polymorphism. Relations to plasma lipoprotein changes associated with abdominal visceral obesity. Arterioscler Thromb 14:527-33, 1994. |
| 1. Pouliot MC, Després J-P, Lemieux S, Moorjani S, Bouchard C, Tremblay A, Nadeau A, Lupien PJ. Waist circumference and abdominal sagittal diameter: best simple anthropometric indexes of abdominal visceral adipose tissue accumulation and related cardiovascular risk in men and women. Am J Cardiol 73:460-8, 1994. |
| 1. Lemieux S, Després J-P, Moorjani S, Nadeau A, Thériault G, Prud’homme D, Tremblay A, Bouchard C, Lupien PJ. Are gender differences in cardiovascular disease risk factors explained by the level of visceral adipose tissue? Diabetologia 37:757-64, 1994. |
| 1. Oppert JM, Vohl MC, Chagnon M, Dionne FT, Cassard-Doulcier AM, Ricquier D, Perusse L, Bouchard C. DNA polymorphism in the uncoupling protein (UCP) gene and human body fat. Int J Obes 18:526-31, 1994. |
| 1. Vohl MC, Dionne FT, Perusse L, Dériaz O, Chagnon M, Bouchard C. Relation between *BglII* polymorphism in 3b-hydroxysteroid dehydrogenase gene and adipose tissue distribution in humans. Obes Res 2:444-9, 1994. |
| 1. Rice T, Province M, Perusse L, Bouchard C, Rao DC. Cross-trait familial resemblance for body fat and blood pressure: Familial correlations in the Québec Family Study. Am J Hum Genet 55:1019-29, 1994. |
| 1. Bouchard C. Recent advances in the molecular and genetic basis of human obesity. In: Ditschuneit H, Gries FA, Hauner H, Schusdziarra V, Wechsler JG, editors.  Obesity in Europe 1993. Proceedings of the 5th European Congress on Obesity. London, England: John Libbey & Company Ltd; 1994. p. 1-8 |
| 1. Oppert JM, Tourville J, Chagnon M, Mauriège P, Dionne FT, Perusse L, Bouchard C. DNA polymorphisms in the a2 and b2-adrenoceptor genes and regional fat distribution in humans: association and linkage studies. Obes Res 3:249-55, 1995. |
| 1. St-Amand J, Després J-P, Lemieux S, Lamarche B, Moorjani S, Prud’homme D, Bouchard C, Lupien PJ. Does lipoprotein or hepatic lipase activity explain the protective lipoprotein profile of premenopausal women? Metabolism 44:491-8, 1995. |
| 1. Tremblay A, Wouters E, Wenker M, St-Pierre S, Bouchard C, Després J-P. Alcohol and a high-fat diet: a combination favoring overfeeding. Am J Clin Nutr 62:639-44, 1995. |
| 1. Vohl MC, Lamarche B, Moorjani S, Prud’homme D, Nadeau A, Bouchard C, Lupien PJ, Després J-P. The lipoprotein lipase *Hin*dIII polymorphism modulates plasma triglyceride levels in visceral obesity. Arterioscler Thromb Vasc Biol 15:714-20, 1995. |
| 1. Song TMK, Thériault G, Prud’homme D, Malina RM, Bouchard C. Relationships of physical fitness, fatness, and lifestyle indicators with blood iron in children and adults. Am J Hum Biol 7:631-41, 1995. |
| 1. Buemann B, Tremblay A, Bouchard C. Social class interacts with the association between macronutrient intake and subcutaneous fat. Int J Obes 19:770-5, 1995. |
| 1. Lemieux S, Prud’homme D, Moorjani S, Tremblay A, Bouchard C, Lupien PJ, Després J-P. Do elevated levels of abdominal visceral adipose tissue contribute to age-related differences in plasma lipoprotein concentrations in men? Atherosclerosis 118:155-64, 1995. |
| 1. Tremblay A, Buemann B, Thériault G, Bouchard C. Body fatness in active individuals reporting low lipid and alcohol intake. Eur J Clin Nutr 49:824-31, 1995. |
| 1. Rice T, Bouchard C, Perusse L, Rao DC. Familial clustering of multiple measures of adiposity and fat distribution in the Québec Family Study: A trivariate analysis of percent body fat, body mass index, and trunk-to-extremity skinfold ratio. Int J Obes 19:902-8, 1995. |
| 1. Shephard RJ, Bouchard C. Relationship between perceptions of physical activity and health-related fitness. J Sports Med Phys Fitness 35:149-58, 1995. |
| 1. Bouchard C. Genetics of body fat content. In: Angel A, Anderson H, Bouchard C, Lau D, Leiter L, Mendelson R, editors. Progress in Obes Res: 7. Proceedings of the 7th International Congress on Obesity. London, England: John Libbey & Company Ltd; 1996. p. 33-41. |
| 1. Perusse L, Després J-P, Lemieux S, Rice T, Rao DC, Bouchard C. Familial aggregation of abdominal visceral fat level: results from the Quebec Family Study. Metabolism 45:378-82, 1996. |
| 1. Bouchard C. Long-term programming of body size. Sixteenth Marabou Symposium Early Nutrition and Lifelong Health. Nutr Rev 54:S8-S16, 1996. |
| 1. Bouchard C, Rice T, Lemieux S, Després J-P, Perusse L, Rao DC. Major gene for abdominal visceral fat area in the Québec Family Study. Int J Obes 20:420-7, 1996. |
| 1. Rice T, Perusse L, Bouchard C, Rao DC. Familial clustering of abdominal visceral fat and total fat mass: The Québec Family Study. Obes Res 4:253-61, 1996. |
| 1. Bouchard C. Genetic epidemiology, association, and sib-pair linkage: Results from the Québec Family Study. In: Bray GA, Ryan DH, editors. Molecular and genetic aspects of obesity. Vol. 5. Pennington Center Nutrition Series. Baton Rouge, LA: Louisiana State University Press; 1996. p. 470-81. |
| 1. Shephard RJ, Bouchard C. Associations between health behaviours and health related fitness. Br J Sports Med 30:94-101, 1996. |
| 1. Couillard C, Larmarche B, Tchernof A, Prud’homme D, Tremblay A, Bouchard C, Moorjani S, Nadeau A, Lupien PJ, Després J-P. Plasma high-density lipoprotein cholesterol but not apolipoprotein A-I is a good correlate of the visceral obesity-insulin resistance dyslipidemic syndrome. Metabolism 45:882-8, 1996. |
| 1. Li Z, Rice T, Perusse L, Bouchard C, Rao DC. Familial aggregation of subcutaneous fat patterning: Principal components of skinfolds in the Québec Family Study. Am J Hum Biol 8:535-42, 1996. |
| 1. Rice T, Tremblay A, Dériaz O, Perusse L, Rao DC, Bouchard C. A major gene for resting metabolic rate unassociated with body composition: results from the Québec Family Study. Obes Res 4:441-9, 1996. |
| 1. Vohl MC, Tchernof A, Dionne FT, Moorjani S, Prud’homme D, Bouchard C, Nadeau A, Lupien PJ, Després J-P. The ApoB-100 gene *Eco*RI polymorphism influences the relationship between features of the insulin resistance syndrome and the Hyper-ApoB and dense LDL phenotype in men. Diabetes 45:1405-11, 1996. |
| 1. Gagnon J, Mauriège P, Roy S, Sjöström D, Chagnon YC, Dionne FT, Oppert JM, Perusse L, Sjöström L, Bouchard C. The Trp64Arg mutation of the b3 adrenergic receptor gene has no effect on obesity phenotypes in the Québec Family Study and Swedish Obese Subjects cohorts. J Clin Invest 98:2086-93, 1996. |
| 1. Rice T, Tremblay A, Dériaz O, Perusse L, Rao DC, Bouchard C. Genetic pleiotropy for resting metabolic rate with fat-free mass and fat mass: The Québec Family Study. Obes Res 4:125-31, 1996. |
| 1. Lemieux S, Prud’homme D, Bouchard C, Tremblay A, Després J-P. A single threshold value of waist girth identifies normal-weight and overweight subjects with excess visceral adipose tissue. Am J Clin Nutr 64:685-93, 1996. |
| 1. Couillard C, Lemieux S, Moorjani S, Lupien PJ, Thériault G, Prud’homme D, Tremblay A, Bouchard C, Després J-P. Associations between 12-year changes in body fatness and lipoprotein-lipid levels in men and women of the Québec Family Study. Int J Obes 20:1081-8, 1996. |
| 1. Rice T, Nadeau A, Perusse L, Bouchard C, Rao DC. Familial correlations in the Québec family study: cross-trait familial resemblance for body fat with plasma glucose and insulin. Diabetologia 39:1357-64, 1996. |
| 1. Malina RM, Katzmarzyk PT, Song TMK, Thériault G, Bouchard C.  Somatotype and cardiovascular risk factors in healthy adults. Am J Hum Biol 9:11-9, 1997. |
| 1. Chagnon YC, Perusse L, Lamothe M, Chagnon M, Nadeau A, Dionne FT, Gagnon J, Chung WK, Leibel RL, Bouchard C. Suggestive linkages between markers on human 1p32-p22 and body fat and insulin levels in the Quebec Family Study. Obes Res 5:115-21, 1997. |
| 1. Vohl MC, Lamarche B, Bergeron J, Moorjani S, Prud'homme D, Nadeau A, Tremblay A, Lupien PJ, Bouchard C, Després J-P. The *Msp*I polymorphism of the apolipoprotein A-II gene as a modulator of the dyslipidemic state found in visceral obesity. Atherosclerosis 128:183-90, 1997. |
| 1. Bouchard C. Human variation in body mass: evidence for a role of the genes. Nutr Rev 55:S21-S30, 1997. |
| 1. Buemann B, Vohl MC, Chagnon M, Chagnon YC, Gagnon J, Perusse L, Dionne F, Després J-P, Tremblay A, Nadeau A, Bouchard C. Abdominal visceral fat is associated with a *Bcl*I restriction fragment length polymorphism at the glucocorticoid receptor gene locus. Obes Res 5:186-92, 1997. |
| 1. Lembertas AV, Perusse L, Chagnon YC, Fisler JS, Warden CH, Purcell-Huynh DA, Dionne FT, Gagnon J, Nadeau A, Lusis AJ, Bouchard C. Identification of an obesity quantitative trait locus on mouse chromosome 2 and evidence of linkage to body fat and insulin on the human homologous region 20q. J Clin Invest 100:1240-7, 1997. |
| 1. Tchernof A, Labrie F, Bélanger A, Prud'homme D, Bouchard C, Tremblay A, Nadeau A, Després J-P . Relationships between endogenous steroid hormone, sex hormone-binding globulin and lipoprotein levels in men: contribution of visceral obesity, insulin levels and other metabolic variables. Atherosclerosis. 133:235-44, 1997. |
| 1. Bouchard C, Perusse L, Chagnon YC, Warden C, Ricquier D. Linkage between markers in the vicinity of the uncoupling protein 2 gene and resting metabolic rate in humans. Hum Mol Genet 6:1887-9, 1997. |
| 1. Bouchard C. Genetic determinants of regional fat distribution. Hum Reprod 12 (Suppl 1):1-5, 1997. |
| 1. Chagnon YC, Chen WJ, Perusse L, Chagnon M, Nadeau A, Wilkison WO, Bouchard C. Linkage and association studies between the melanocortin receptors 4 and 5 genes and obesity-related phenotypes in the Québec Family Study. Mol Med 3:663-73, 1997. |
| 1. Perusse L, Rice T, Després J-P, Rao DC, Bouchard C. Cross-trait familial resemblance for body fat and blood lipids: familial correlations in the Quebec Family Study. Arterioscler Thromb Vasc Biol 17:3270-7, 1997. |
| 1. Gu C, Rice T, Perusse L, Bouchard C, Rao DC. Principal components analysis of morphological measures in the Québec Family Study: Familial correlations. Am J Hum Biol  9:725-33, 1997. |
| 1. Tchernof A, Labrie F, Belanger A, Prud’homme D, Bouchard C, Tramblay A, Nadeau A, Depres J-P. Androstane-3a, 17 b-diol glucuronide as a steroid correlate of visceral obesity in men. J Clin Endocrin Metab 82: 1528-34, 1997. |
| 1. Doucet E, Alméras N, White MD, Després J-P, Bouchard C, Tremblay A. Dietary fat composition and human adiposity. Eur J Clin Nutr  52:2-6, 1998. |
| 1. Katzmarzyk PT, Malina RM, Song TMK, Thériault G, Bouchard C. Physique and echocardiographic dimensions in children, adolescents and young adults. Ann Hum Biol  25:145-57, 1998. |
| 1. Katzmarzyk PT, Malina RM, Song TMK, Bouchard C. Somatotype and indicators of metabolic fitness in youth. Am J Hum Biol  10:341-50, 1998. |
| 1. Tremblay A, Drapeau V, Doucet E, Alméras N, Després J-P, Bouchard C. Fat balance and ageing: results from the Québec Family Study. Br J Nutr  79:413-8, 1998. |
| 1. Katzmarzyk PT, Malina RM, Song TMK, Bouchard C. Television viewing, physical activity, and health-related fitness of youth in the Québec Family Study. J Adolesc Health  23: 318-25, 1998. |
| 1. Katzmarzyk PT, Malina RM, Song TMK, Bouchard C. Physical activity and health-related fitness in youth: a multivariate analysis. Med Sci Sports Exerc  30:709-14, 1998. |
| 1. Perusse L, Chagnon YC, Rice T, Rao DC, Bouchard C. L'épidémiologie génétique et la génétique moléculaire de l'obésité: les enseignements de l'étude des familles de Québec. Médecine/Sciences  14:914-24, 1998. |
| 1. Borecki IB, Blangero J, Rice T, Perusse L, Bouchard C, Rao DC. Evidence for at least two major loci influencing human fatness. Am J Hum Genet  63:831-8, 1998. |
| 1. Katzmarzyk PT, Perusse L, Bouchard C. Genetics of abdominal visceral fat levels. Am J Hum Biol  11:225-35, 1999. |
| 1. Rice T, Perusse L, Bouchard C, Rao DC. Familial aggregation of body mass index and subcutaneous fat measures in the longitudinal Québec Family Study. Genet Epidemiol 16:316-34, 1999. |
| 1. Chagnon YC, Chung WK, Perusse L, Chagnon M, Leibel RL, Bouchard C. Linkages and associations between the leptin receptor (LEPR) gene and human body composition in the Québec Family Study. Int J Obes  23:278-86, 1999. |
| 1. Dionne I, Després J-P, Bouchard C, Tremblay A. Gender difference in the effect of body composition on energy metabolism. Int J Obes  23:312-9, 1999. |
| 1. Bouchard C, Perusse L, Chagnon YC. Genes and human obesity. In: Bray  GA and Ryan DH, editors. Nutrition, Genetics, and Obesity. Vol. 9. Pennington Center Nutrition Series. Baton Rouge, LA: Louisiana State University Press; 1999. p. 25-45. |
| 1. Rankinen T, Perusse L, Dériaz O, Thériault G, Chagnon M, Nadeau A, Bouchard C. Linkage of the Na,K-ATPase a2 and b1 genes with resting and exercise heart rate and blood pressure: cross-sectional and longitudinal observations from the Quebec Family Study. J Hypertens  17:339-49, 1999. |
| 1. St-Amand J, Prud'homme D, Moorjani S, Nadeau A, Tremblay A, Bouchard C, Lupien PJ, Després J-P . Apolipoprotein E polymorphism and the relationships of physical fitness to plasma lipoprotein-lipid levels in men and women. Med Sci Sports Exerc  31:692-7, 1999. |
| 1. Katzmarzyk P, Malina RM, Song TMK, Bouchard C. Physique, subcutaneous fat, adipose tissue distribution, and risk factors in the Québec Family Study. Int J Obes  23:476-84, 1999. |
| 1. Katzmarzyk PT, Rankinen T, Perusse L, Dériaz O, Tremblay A, Borecki I, Rao DC, Bouchard C. Linkage and association of the sodium potassium-adenosine triphosphatase a2 and b1 genes with respiratory quotient and resting metabolic rate in the Québec Family Study. J Clin Endocrinol Metab  84:2093-7, 1999. |
| 1. Rivera MA, Perusse L, Gagnon J, Dionne FT, Leon AS, Rao DC, Skinner JS, Wilmore JH, Sjöström L, Bouchard C. A mitochondrial DNA D-loop polymorphism and obesity in three cohorts of women. Int J Obes  23: 666-8, 1999. |
| 1. Eisenmann JC, Katzmarzyk PT, Thériault G, Song TMK, Malina RM, Bouchard C. Physical activity and pulmonary function in youth: The Québec Family Study. Pediatr Exerc Sci 11:208-17, 1999. |
| 1. Vohl M-C, Lamarche B, Pascot A, Leroux G, Prud'homme D, Bouchard C, Nadeau A, Despres J-P. Contribution of the cholesteryl ester transfer protein gene TaqlB polymorphism to the reduced plasma HDL-cholesterol levels found in abdominal obese men with the features of the insulin resistance syndrome. Int J Obes 23:918-25, 1999. |
| 1. Pascot A, Lemieux S, Lemieux I, Prud'homme D, Tremblay A, Bouchard C, Nadeau A, Couillard C, Tchernof A, Bergeron J, and Despres J-P. Age-related increase in visceral adipose tissue and body fat and the metabolic risk profile of premenopausal women. Diabetes Care 22:1471-8, 1999. |
| 1. Lemieux I, Pascot A, Tchernof A, Bergeron J, Prud'homme D, Bouchard C, Despres J-P. Visceral adipose tissue and low-density lipoprotein particle size in middle-aged versus young men. Metabolism 48:1322-7, 1999. |
| 1. Rankinen T, Kim S-Y, Perusse L, Despres J-P, Bouchard C.  The prediction of abdominal visceral fat level from body composition and anthropometry: ROC analysis.  Int J Obes  23:801-9, 1999. |
| 1. Katzmarzyk PT, Malina RM, Bouchard C.  Physical activity, physical fitness, and coronary heart disease risk factors in youth: The Quebec Family Study.  Prev Med  29:555-62, 1999. |
| 1. Dionne I, Almeras N, Bouchard C, Tremblay A.  The association between vigorous physical activities and fat deposition in male adolescents. Med Sci Sports Exerc 32:392-5, 2000. |
| 1. Chagnon YC, Borecki IB, Perusse L, Roy S, Lacaille M, Chagnon M, Ho-Kim MA, Rice T, Province MA, Rao DC, Bouchard C.  Genome-Wide Search for Genes Related to the Fat-Free Body Mass in the Quebec Family Study.  Metabolism 49:203-7, 2000. |
| 1. Rosmond R, Chagnon YC, Holm G, Chagnon M, Perusse L, Lindell K, Carlsson B, Bouchard C, and Bjorntorp P. A Glucocorticoid Receptor Gene Marker is Associated with Abdominal Obesity, Leptin, and Dysregulation of the Hypothalamic-Pituitary-Adrenal Axis. Obes Res 8:211-8, 2000. |
| 1. Rice T, Rankinen T, Province MA, Chagnon YC, Perusse L, Borecki IB, Bouchard C, Rao DC. Genome-Wide Linkage Analysis of Systolic and Diastolic Blood Pressure, The Quebec Family Study. Circulation 102:1956-63 2000. |
| 1. Ukkola O, Rankinen T, Weisnagel SJ, Sun G, Perusse L, Chagnon YC, Despres JP, Bouchard C.  Interactions Among the alpha 2-, beta 2-, and beta 3-Adrenergic Receptor Genes and Obesity-Related Phenotypes in the Quebec Family Study.  Metabolism  49:1063-70, 2000. |
| 1. Katzmarzyk PT, Perusse L, Tremblay A, Bouchard C. No association between resting metabolic rate or respiratory exchange ratio and subsequent changes in body mass and fatness: 5 ½ year follow up of the Quebec Family Study. Eur J Clin Nutr. 54:610-4, 2000. |
| 1. Rice T, Rao R, Perusse L, Bouchard C and Rao DC. Tracking of Familial Resemblance for Resting Blood Pressure over Time in the Quebec Family Study.  Hum Biol 72:415-31, 2000. |
| 1. Doucet E, St-Pierre S, Almeras N, Mauriege P, Despres JP, Richard D, Bouchard C and Tremblay A. Fasting insulin levels influence plasma leptin levels independently from the contribution of adiposity: evidence from both a cross-sectional and an intervention study. J Clin Endocrinol Metab 85: 4231-7, 2000. |
| 1. Eisenmann JC, Katzmarzyk PT, Theriault G, Song TMK, Malina RM, Bouchard C.   Cardiac dimensions, physical activity, and submaximal working capacity in youth of the Quebec Family Study. Eur J Appl Physiol  81:40-6, 2000. |
| 1. Campbell PT, Katzmarzyk PT, Malina RM, Rao DC, Perusse L, Bouchard C. Prediction of physical activity and PWC150 in young adulthood from childhood and adolescence with consideration of parental measures. Am J Hum Biol 13:190-6, 2001. |
| 1. Ukkola O, Perusse L, Weisnagel SJ, Bergeron J, Despres J-P, Rao DC, and Bouchard C. Interactions Among the Glucocorticoid Receptor, Lipoprotein Lipase and Adrenergic Receptor Genes and Plasma Insulin and Lipid Levels in the Quebec Family Study. Metabolism 50:246-52, 2001. |
| 1. Yoshioka M, Doucet E, St-Pierre S, Almeras N, Richard D, Labrie A, Despres JP, Bouchard C, and Tremblay A. Impact of high-intensity exercise on energy expenditure, lipid oxidation and body fatness. Int J Obes 25:332-9, 2001. |
| 1. Katzmarzyk P, Perusse L, Malina RM, Bergeron J, Despres JP, and Bouchard C. Stability of indicators of the metabolic syndrome from childhood and adolescence to young adulthood: the Quebec Family Study. J Clin Epidemiol 54:190-5, 2001. |
| 1. Perusse L, Rice T, Chagnon YC, Despres JP, Lemieux S, Roy S, Lacaille M, Ho-Kim MY, Chagnon M, Province MA, Rao DC, and Bouchard C. A genome-wide scan for abdominal fat assessed by computed tomography in the Quebec Family Study. Diabetes 50:614-21, 2001. |
| 1. Weisnagel SJ, Rankinen T, Nadeau A, Rao DC, Chagnon YC, Perusse L, and Bouchard C. Decreased fasting and oral glucose stimulated c-peptide in nondiabetic subjects with sequence variants in the sulfonylurea receptor 1 gene. Diabetes 50:697-702, 2001. |
| 1. Campbell PT, Katzmarzyk PT, Malina RM, Rao DC, Perusse L and Bouchard C.  Stability of adiposity phenotypes from childhood and adolescence into young adulthood with contribution of parental measures.  Obes Res 9(7):394-400, 2001. |
| 1. Doucet E, St-Pierre S, Almeras N, Despres J-P, Bouchard C, and Tremblay A.  Evidence for the existence of adaptive thermogenesis during weight loss.  British J Nutr 85:715-23, 2001. |
| 1. Seidell JC, Perusse L, Despres J-P, and Bouchard C.  Waist and hip circumferences have independent and opposite effects on cardiovascular disease risk factors: the Quebec Family Study.  Am J Clin Nutr 74:315-21, 2001. |
| 1. Ukkola O, Garenc C, Perusse L, Bergeron J, Despres J-P, Rao DC and Bouchard C.  Genetic variation at the lipoprotein lipase locus and plasma lipoprotein and insulin levels in the Quebec Family Study. Arteriosclerosis 158: 199-206, 2001. |
| 1. Ukkola O, Perusse L, Chagnon YC, Despres J-P and Bouchard C.  Interactions among the glucocorticoid receptor, lipoprotein lipase and adrenergic receptor genes and abdominal fat in the Quebec Family Study. Int J Obes 25: 1332-9, 2001. |
| 1. Lanouette C-M, Giacobino J-P, Perusse L, Lacaille M, Yvon C, Chagnon M, Kuhne F, Bouchard C, Muzzin P and Chagnon YC.  Association between uncoupling protein 3 gene and obesity-related phenotypes in the Quebec Family Study.  Mol Med 7(7): 433-41, 2001. |
| 1. Heo M, Leibel RL, Boyer BB, Chung WK, Koulu M, Karvonen MK, Pesonen U, Rissanen A, Laakso M, Uusitupa MIJ, Chagnon Y, Bouchard C, Donohoue PA, Burns TL, Shuldiner AR, Silver K, Andersen RE, Pedersen O, Echwald S, Sorensen TIA, Behn P, Permutt MA, Jacobs KB, Elston RC, Hoffman DJ and Allison DB. Pooling analysis of genetic data: the association of leptin receptor (LEPR) polymorphisms with variables related to human adiposity. Genetics 159:1163-1178, 2001. |
| 1. Tanaka S, Togashi K, Rankinen T, Perussse L, Leon AS, Rao DC, Skinner JS, Wilmore JH and Bouchard C.  Is adiposity at normal body weight relevant for cardiovascular disease risk? Int J Obes 26: 176-83, 2002. |
| 1. Heo M, Leibel RL, Fontaine KR, Boyer BB, Chung WK, Koulu M, Karvonen MK, Pesonen U, Rissanen A, Laasko M, Wwsitupa MIJ, Chagnon Y, Bouchard C, Donohoue PA, Burns TL, Shuldiner AR, Silver K, Andersen RE, Pedersen O, Schwald S, Sorensen TIA, Behn P, Permutt MA, Jacobs KB, Elston RC, Hoffman DJ, Gropp E and Allison DB. A meta-analytic investigation of linkage and association of common leptin receptor (LEPR) polymorphisms with body mass index and waist circumference. Int J Obes 26(5):640-6, 2002. |
| 1. Simonen RL, Perusse L, Rankinen T, Rice T, Rao DC and Bouchard C.  Familial aggregation of physical activity levels in the Quebec family study.  Med Sci Sports Exerc  34(7): 1137-42, 2002. |
| 1. Ukkola O, Ravussin E, Jacobson P, Perusse L, Rankinen T, Tschop M, Heiman ML, Leon AS, Rao DC, Skinner JS, Wilmore JH, Sjostrom L and Bouchard C.  Role of ghrelin polymorphisms in obesity based on three different studies. Obes Res 10; 782-91, 2002. |
| 1. Boucher N, Lanouette CM, Larose M, Perusse L, Bouchard C, and Chagnon YC. A +2138InsCAGACC polymorphism of the melanocortin receptor 3 gene is associated in human with fat level and partitioning in interaction with body corpulence.  Molec Med 8(3): 158-65, 2002. |
| 1. Bosse Y, Perusse L, Despres J-P, Lamarche B, Chagnon YC, Rice T, Rao DC, Bouchard C and Vohl M-C.  Evidence for a major quantitative trait locus on chromosome 17q21 affecting low-density lipoprotein peak particle diameter. Circulation 107: 2361-8, 2003. |
| 1. Imbeault P, Prins JB, Stolic J, Russell AW, O’Moore-Sullivan T, Despres J-P, Bouchard C and Tremblay A. Aging per se does not influence glucose homeostasis. Diabetes Care 26:480-484, 2003. PMID: 12547885 |
| 1. Robitaille J, Despres JP, Perusse L, Vohl MC.  The PPAR-gamma P12A polymorphism modulates the relationship between dietary fat intake and components of the metabolic syndrome: results from the Quebec Family Study. Clin Genet 63(2):109-16, 2003. |
| 1. Simonen RL, Rankinen T, Perusse L, Leon AS, Skinner JS, Wilmore JH, Rao DC and Bouchard C.  A dopamine D2 receptor gene polymorphism and physical activity in two family studies. Physiol Behav 78: 751-7, 2003. |
| 1. Jacqmain M, Doucet E, Despres J-P, Bouchard C and Tremblay A.  Calcium intake, body composition, and lipoprotein-lipid concentrations in adults.  Am J Clin Nutr 77:1448-52, 2003. |
| 1. Katzmarzyk PT, Tremblay A, Perusse L, Despres J-P and Bouchard C.  The utility of the international child and adolescent overweight guidelines for predicting coronary heart disease risk factors.  J Clin Epidemiol 56: 456-62, 2003. |
| 1. Doucet E, Imbeault P, St-Pierre S, Almeras N, Mauriege P, Despres J-P, Bouchard C and Tremblay A.  Greater than predicted decrease in energy expenditure during exercise after body weight loss in obese men.  Clin Sci; 105: 89-98, 2003. |
| 1. Drapeau V, Provencher V, Lemieux, Despes J-P, Bouchard C and Tremblay A.  Do 6-y changes in eating behaviors predict changes in body weight?  Results from the Quebec Family Study.  Int J Obes 27: 808-14, 2003. |
| 1. Bosse Y, Despres J-P, Bouchard C, Perusse L and Vohl M-C.  The peroxisome proliferator-activated receptor α L162V mutation is associated with reduced adiposity.  Obes Res 11:809-16, 2003. |
| 1. Tremblay A, Bouchard L, Bouchard C, Despres J-P, Drapeau V and Perusse L.  Long-term adiposity changes are related to a glucocorticoid receptor polymorphism in young females.  J Clin Endocrinol Metab 88: 3141-5, 2003. |
| 1. Eisenmann JC, Katzmarzyk PT, Perusse L, Bouchard C and Malina RM. Estimated daily energy expenditure and blood lipids in adolescents: The Quebec Family Study.  J Adoles Health 33:147-53, 2003. |
| 1. Simonen RL, Rankinen T, Perusse L, Rice T, Rao DC, Chagnon Y and Bouchard C.  Genome-wide linkage scan for physical activity levels in the Quebec Family Study.  Med Sci Sports Exerc 35(8): 1355-9, 2003. |
| 1. Perusse L, Chagnon YC and Bouchard C.  Genome-wide scan studies of obesity-related phenotypes in the Quebec and HERITAGE Family Studies. In: Progress in Obes Res: 9. Proceedings of the 9th International Congress on Obesity, G. Medeiros-Neto, A Halpern and C. Bouchard, editors.  Sao Paulo, Brazil: John Libbey & Company Ltd; 2003. p. 333-7. |
| 1. Katzmarzyk PT, Despres J-P and Bouchard C. Body types and health.  In: Progress in Obes Res: 9. Proceedings of the 9th International Congress on Obesity, G. Medeiros-Neto, A. Halpern and C. Bouchard, editors.  Sao Paulo, Brazil: John Libbey & Company Ltd; 2003. p. 644-8. |
| 1. Perusse L and Bouchard C. Bases genetiques de l’obesite familiale au Quebec.  Medecine/Sciences 19:937-42, 2003. |
| 1. Bosse Y, Vohl M-C, Despres J-P, Lamarche B, Rice T, Rao DC, Bouchard C and Perusse L. Heritability of LDL peak particle diameter in the Quebec Family Study. Genet Epidemiol 25:375-81, 2003. |
| 1. Tanaka S, Togahi K, Rankinen T, Perusse L, Leon AS, Rao DC, Skinner JS, Wilmore JH, Depres J-P, and Bouchard C.  Sex differences in the relationship of abdominal fat to cardiovascular disease risk among normal-weight white subjects. Int J Obes Relat Metab Disord 28: 320-3, 2004. |
| 1. Paradis M-E, Couture P, Bosse Y, Despres J-P, Perussse L, Bouchard C, Vohl M-C, and Lamarche B.   The T111l mutation in the EL gene modulates the impact of dietary fat on the HDL profile in women.  J Lipid Res 44: 1902-8, 2003. |
| 1. Bosse Y, Chagnon YC, Despres J-P, Rice T, Rao DC, Bouchard C, Perusse L and Vohl M-C. Genome-wide linkage scan reveals multiple susceptibility loci influencing lipid and lipoprotein levels in the Quebec Family Study.  J Lipid Res 45: 419-26, 2004. |
| 1. Provencher V, Drapeau V, Tremblay A, Despres J-P, Bouchard C and Lemieux S.  Eating behaviors, dietary profile and body composition according to dieting history in men and women of the Quebec Family Study.  Br J Nutr 91: 997-1004, 2004. |
| 1. Drapeau V, Despres J-P, Bouchard C, Allard L, Fournier G, Leblanc C and Tremblay A.  Modifications in food-group consumption are related to long-term body-weight changes.  Am J Clin Nutr 80:29-37, 2004. |
| 1. Bouchard L, Drapeau V, Provencher V, Lemieux S, Chagnon Y, Rice T, Rao DC, Vohl M-C, Tremblay, A, Bouchard C and Perusse L.  Neuromedin b: a strong candidate gene linking eating behaviors and susceptibility to obesity.  Am J Clin Nutr 80: 1478-86, 2004. |
| 1. Bosse Y, Chagnon YS, Despres J-P, Rice T, Rao DC, Bouchard C, Perusse L, and Vohl M-C.  Compendium of genome-wide scans of lipid-related phenotypes: adding a new genome-wide search of apolipoprotein levels.  J Lipid Res  45: 2174-84, 2004. |
| 1. Bouchard L, Weisnagel SJ, Engert JC, Hudson TJ, Bouchard C, Vohl M-C, and Perusse L.  Human resistin gene polymorphism is associated with visceral obesity and fasting and oral glucose stimulated C-peptide in the Quebec Family Study.  J Endocrinol Invest 27: 1003-9, 2004. |
| 1. Loos RJF, Rankinen T, Tremblay A, Perusse L, Chagnon Y, and Bouchard C.  Melanocortin-4 receptor gene and physical activity in the Quebec Family Study.  Int J Obes 29:420-8, 2005. |
| 1. Eisenmann JC, Katzmarzyk PT, Perusse L, Tremblay A, Despres J-P, Bouchard C. Aerobic fitness, body mass index, and CVD risk factors among adolescents: the Quebec family study. Int J Obes 29(9):1077-83, 2005. |
| 1. Santoro N, Rankinen T, Perusse L, Loos RJ, Bouchard C. MC4R marker associated with stature in children and young adults: a longitudinal study. J Pediatr Endocrinol Metab 18(9): 859-63, 2005. |
| 1. Boule NG, Bouchard C, Tremblay A. Physical fitness and the metabolic syndrome in adults from the Quebec Family Study. Can J Appl Physiol 30(2):140-56, 2005. |
| 1. Bouchard L, Mauriege P, Vohl MC, Bouchard C, Perusse L. Plasminogen-activator inhibitor-1 polymorphisms are associated with obesity and fat distribution in the Quebec Family Study: evidence of interactions with menopause. Menopause 12(2):136-43, 2005. |
| 1. Katzmarzyk P and Bouchard C. Genetic influences on human body composition.  IN: Heymsfield SB, Lohman TG, Wang Z, and Going SB (eds.). Human Body Composition. Human Kinetics, 2nd Edition, chap 16; 2005. pp 243-57. |
| 1. Blouin K, Despres J-P, Couillard C, Tremblay A, Prud’homme D, Bouchard C, Tchernof A. Contribution of age and declining androgen levels to features of the metabolic syndrome in men. Metabolism 54:1034-40, 2005. PMID: 16092053 |
| 1. Bosse Y, Feitosa MF, Despres JP, Lamarche B, Rice T, Rao DC, Bouchard C, Perusse L, Vohl MC.  Detection of a major gene effect for LDL peak particle diameter and association with apolipoprotein H gene haplotype.  Atherosclerosis 182(2): 231-9, 2005. |
| 1. Provencher V, Perusse L, Bouchard L, Drapeau V, Bouchard C, Rice T, Rao DC, Tremblay A, Despres JP, Lemieux S.  Familial resemblance in eating behaviors in men and women from the Quebec Family Study.  Obes Res 13(9): 1624-9, 2005. |
| 1. Bosse Y, Bouchard L, Despres JP, Bouchard C, Perusse L, Vohl MC.  Haplotypes in the phospholipids transfer protein gene are associated with obesity-related phenotypes: the Quebec Family Study.  Int J Obes 29: 1338-45, 2005. |
| 1. Loos RJ, Rankinen T, Chagnon Y, Tremblay A, Perusse L, Bouchard C.  Polymorphisms in the leptin receptor genes in relation to resting metabolic rate and respiratory quotient in the Quebec Family Study.  Int J Obes 30(1): 183-90, 2006. |
| 1. Alfonzo-Gonzalez G, Doucet E, Bouchard C, Tremblay A.  Greater than predicted decrease in resting energy expenditure with age: cross-sectional and longitudinal evidence.  Eur J Clin Nutr 60(1): 18-24, 2006. |
| 1. Desilets MC, Garrel D, Couillard C, Tremblay A, Despres JP, Bouchard C, Delisle H. Ethnic differences in body composition and other markers of cardiovascular disease risk: study in matched Haitian and white subjects from Quebec. Obesity 14(6): 1019-27, 2006. |
| 1. Bailey SD, Loredo-Osti JC, Lepage P, Faith J, Fontaine J, Desbiens KM, Hudson TJ, Bouchard C, Gaudet D, Perusse L, Vohl MC, Engert JC. Common polymorphisms in the promoter of the visfatin gene (PBEF1) influence plasma insulin levels in a French-Canadian population. Diabetes 55(10): 2896-902, 2006. |
| 1. Aissani B, Perusse L, Lapointe G, Chagnon YC, Bouchard L, Walts B, Bouchard C.  A quantitative trait locus for body fat on chromosome 1q43 in French Canadians: linkage and association studies.  Obesity 14(9): 1605-15, 2006. |
| 1. Jacobson P, Rankinen T, Tremblay A, Perusse L, Chagnon YC, Bouchard C. Resting metabolic rate and respiratory quotient: results from a genome-wide scan in the Quebec Family Study. Am J Clin Nutr 84: 1527-33, 2006. |
| 1. Loos RJF, Ruchat S, Rankinen T, Tremblay A, Perusse L, Bouchard C. Adiponectin and adiponectin receptor gene variants in relation to resting metabolic rate, respiratory quotient, and adiposity-related phenotypes in the Quebec Family Study. Am J Clin Nutr 85: 26-34, 2007. |
| 1. Chaput JP, Despres JP, Bouchard C, Tremblay A. Short sleep duration is associated with reduced leptin levels and increased adiposity: results from the Quebec Family Study. Obesity 15(1): 253-61, 2007. |
| 1. Eisenmann JC, Malina RM, Tremblay A, Bouchard C.  Adiposity and cardiac dimensions among 9- to 18-year-old youth: the Quebec Family Study.  J Hum Hypertens 12(2): 114-9, 2007. |
| 1. Choquette A, Bouchard L, Houde A, Bouchard C, Perusse L, Vohl MC.  Associations between USF1 gene variants and cardiovascular risk factors in the Quebec Family Study. Clin Genet 71(3): 245-53, 2007. |
| 1. Bosse Y, Despres JP, Chagnon YC, Rice T, Rao DC, Bouchard C, Perusse L, Vohl MC. Quantitative trait locus on 15q for a metabolic syndrome variable derived from factor analysis. Obesity 15(3): 544-50, 2007. |
| 1. Bouchard L, Tremblay A, Bouchard C, Perusse L.  Contribution of several candidate gene polymorphisms in the determination of adiposity changes: results from the Quebec Family Study.  Int J Obes 31(6): 891-9, 2007. |
| 1. Arsenault B, Lachance D, Lemieux I, Almeras N, Tremblay A, Bouchard C, Perusse L, Despres JP.  Visceral Adipose Tissue Accumulation, Cardiorespiratory Fitness, and Features of the Metabolic Syndrome.  Arch Intern Med  167(14): 1518-25, 2007. |
| 1. Bouchard L, Bouchard C, Chagnon Y, Perusse L.  Evidence of linkage and association with body fatness and abdominal fat on chromosome 15q26.  Obesity 15(8): 2061-70, 2007. |
| 1. Robitaille J, Perusse L, Bouchard C, Vohl MC. Genes, fat intake, and cardiovascular disease risk factors in the Quebec Family Study. Obesity 15(9): 2336-47, 2007. |
| 1. Chaput JP, Després JP, Bouchard C, Tremblay A. Association of sleep duration with type 2 diabetes and impaired glucose tolerance. Diabetologia 2007; 50: 2298-2304. |
| 1. Dolley G, Berthier MT, Lamarche B, Depres JP, Bouchard C, Perusse L, Vohl MC. Influences of the phosphatidylcholine transfer protein gene variants on the LDL peak particle size. Atherosclerosis 195: 297-302, 2007. PMID: 17266964 |
| 1. Loos RJ, Rankinen T, Perusse L, Tremblay A, Despres JP, Bouchard C. Association with Lipin 1 gene polymorphisms with measures of energy and glucose metabolism. Obesity 15(11): 2723-32, 2007. PMID: 18070763 |
| 1. Chaput JP, Tremblay A, Rimm E, Bouchard C, Ludwig D. A novel interaction between dietary composition and insulin secretion: effects on weight gain in the Quebec Family Study. Am J Clin Nutr 87(2): 303-9, 2008. |
| 1. Ruchat SM, Loos RJ, Rankinen T, Vohl MC, Weisnagel SJ, Despres JP, Bouchard C, Perusse L. Associations between glucose tolerance, insulin sensitivity and insulin secretion phenotypes and polymorphisms in adiponectin receptor genes in the Quebec Family Study. Diabet Med 25(4): 400-6, 2008. |
| 1. Do R, Bailey SD, Desbiens K, Belisle A, Montpetit A, Bouchard C, Perusse, L, Vohl MC, Engert JC. Genetic variants of FTO influence adiposity, insulin sensitivity, leptin levels, and resting metabolic rate in the Quebec Family Study. Diabetes 57(4): 1147-50, 2008. |
| 1. Major GC, Doucet E, Jacqmain M, St-Onge M, Bouchard C, Tremblay A. Multivitamin and dietary supplements, body weight and appetite: results from a cross-sectional and a randomized double-blind placebo-controlled study. Br J Nutr 99(4): 1157-67, 2008. |
| 1. Chaput JP, Despres JP, Bouchard C, Tremblay A. The Association Between Sleep Duration and Weight Gain in Adults: A 6-Year Prospective Study from the Quebec Family Study. Sleep 31(4): 517-23, 2008. |
| 1. Boule N, Chaput JP, Doucet E, Richard D, Despres JP, Bouchard C, Tremblay A. Glucose homeostasis predicts weight gain: prospective and clinical evidence. Diabetes Metab Res Rev 24(2): 123-29, 2008. |
| 1. Dolley G, Lamarche B, Despres JP, Bouchard C, Perusse L, Vohl MC. Myeloperoxidase gene sequence variations are associated with low-density-lipoprotein characteristics. J Hum Genet 53(5): 439-46, 2008. |
| 1. Ruchat SM, Despres JP, Weisnagel SJ, Bouchard C, Perusse L. Genome-wide linkage analysis for circulating levels of adipokines and C-reactive protein in the Quebec family study (QFS). J Hum Genet 53(7): 629-36, 2008. |
| 1. Ruchat SM, Girard M, Weisnagel SJ, Bouchard C, Vohl MC, Perusse L. Association between µ-opioid receptor-1 102T>C polymorphism and intermediate type 2 diabetes phenotypes: results from the Quebec Family Study (QFS). Clin Exp Pharmacol Physiol 35(9): 1018-22, 2008. |
| 1. Teran-Garcia M, Rankinen T, Bouchard C. Genes, exercise, growth, and the sedentary, obese child. J Appl Physiol 105(3): 988-1001, 2008. |
| 1. [Choquette AC, Lemieux S, Tremblay A, Chagnon YC, Bouchard C, Vohl MC, Perusse L.](http://www.ncbi.nlm.nih.gov/sites/entrez?orig_db=PubMed&db=pubmed&cmd=Search&term=1142%5Bpage%5D%20AND%20Choquette%20AC%5Bfirst%20author%5D) Evidence of a quantitative trait locus for energy and macronutrient intakes on chromosome 3q27.3: the Quebec Family Study. Am J Clin Nutr  88(4): 1142-8, 2008. |
| 1. Garenc C, Vohl MC, Bouchard C, Perusse L. LIPE C-60G influences the effects of physical activity on body fat and plasma lipid concentrations: The Quebec Family Study. Hum Genomics 3(2):157-68, 2009. |
| 1. Pigeon E, Couillard E, Tremblay A, Bouchard C, Weisnagel SJ, Joanisse DR. Mid-Thigh Subcutaneous Adipose Tissue and Glucose Tolerance in the Quebec Family Study. Obesity Facts 1(6): 310-18, 2008. |
| 1. Cartier A, Cote M, Lemieux I, Perusse L, Tremblay A, Bouchard C, Despres JP. Sex differences in inflammatory markers : what is the contribution of visceral adiposity. Am J Clin Nutr 89(5): 1307-14, 2009. |
| 1. Ruchat SM, Weisnagel SJ, Rankinen T, Bouchard C, Vohl MC, Perusse L. Interaction between HNF4A polymorphisms and physical activity in relation to type 2 diabetes-related traits: Results from the Quebec Family Study. Diabetes Res Clin Pract 84: 211-18, 2009. |
| 1. Dolley G, Lamarche B, Despres JP, Bouchard C, Perusse L, Vohl MC. Phosphoinositide cycle gene polymorphisms affect the plasma lipid profile in the Quebec Family Study. Mol Genet Metab 97(2): 149-54, 2009. |
| 1. Bouchard L, Faucher G, Tchernof A, Deshaies Y, Marceau S, Lescelleur O, Biron S, Bouchard C, Perusse L, Vohl MC. Association of OSBPL11 Gene Polymorphisms with Cardiovascular Disease Risk Factors in Obesity. Obesity 17(7): 1466-72, 2009. |
| 1. Rheaume C, Arsenault B, Belanger S, Perusse L, Tremblay A, Bouchard C, Poirier P, Despres JP. Low Cardiorespiratory Fitness Levels and Elevated Blood Pressure: What is the Contribution of Visceral Adiposity? Hypertension 54(1): 91-7, 2009. |
| 1. Arsenault B, Cartier A, Cote M, Lemieux I, Tremblay A, Bouchard C, Perusse L, Depres JP. Body Composition, Cardiorespiratory Fitness, and Low-Grade Inflammation in Middle-Aged Men and Women. Am J Cardiol 104(2): 240-6, 2009. |
| 1. [Joe B, Saad Y, Lee NH, Frank BC, Achinike OH, Luu TV, Gopalakrishnan K, Toland EJ, Farms P, Yerga-Woolwine S, Manickavasagam E, Rapp JP, Garrett MR, Coe D, Apte SS, Rankinen T, Perusse L, Ehret GB, Ganesh SK, Cooper RS, O’Connor A, Rice T, Weder AB, Chakravarti A, Rao DC, Bouchard C.](http://www.ncbi.nlm.nih.gov/sites/entrez?orig_db=PubMed&db=pubmed&cmd=Search&term=2825%5Bpage%5D%20AND%20Joe%20B%5Bfirst%20author%5D) Positional identification of variants of *Adamts16* linked to inherited hypertension.  Hum Mol Genet 18(15): 2825-38, 2009. |
| 1. Ruchat SM, Elks CE, Loos RJF, Vohl MC, Weisnagel SJ, Rankinen T, Bouchard C, Perusse L. Association between insulin secretion, insulin sensitivity and type 2 diabetes susceptibility variants identified in genome-wide association studies. Acta Diabetol 46(3): 217-26, 2009. |
| 1. Choquette AC, Lemieux S, Tremblay A, Drapeau V, Bouchard C, Vohl MC, Perusse L. GAD2 gene sequence variations are associated with eating behaviors and weight gain in women from the Quebec Family Study. Physiol Behav 98(4): 505-10, 2009. |
| 1. Chaput JP, Despres JP, Bouchard C, Astrup A, Tremblay A. Sleep duration as a risk factor for the development of type 2 diabetes or impaired glucose tolerance: Analyses of the Quebec Family Study. Sleep Med 10(8): 919-24, 2009. |
| 1. Cartier A, Cote M, Lemieux I, Perusse L, Tremblay A, Bouchard C, Despres JP. Age-related differences in inflammatory markers in men: contribution of visceral adiposity. Metabolism 58(10): 1452-58, 2009. |
| 1. Chaput JP, Leblanc C, Perusse L, Despres JP, Bouchard C, Tremblay A. Risk factors for adult overweight and obesity in the Quebec Family Study: Have we been barking up the wrong tree? Obesity 17(10): 1964-70, 2009. |
| 1. Ruchat SM, Weisnagel SJ, Vohl MC, Rankinen T, Bouchard C, Perusse L. Evidence for interaction between PPARG Pro12Ala and PPARGC1A Gly482Ser polymorphisms in determining type 2 diabetes intermediate phenotypes in overweight subjects. Exp Clin Endocrinol Diabetes 117: 455-59, 2009. |
| 1. Ruchat SM, Vohl MC, Weisnagel SJ, Rankinen T, Bouchard C, Perusse L. Combining genetic markers and clinical risk factors improves the risk assessment of impaired glucose metabolism. Ann Med 42(3): 196-206, 2010. |
| 1. Heid IM, Huth C, Loos RJ, …, Wichmann HE. Meta-analysis of the INSIG2 association with obesity including 74,345 individuals: does heterogeneity of estimates relate to study design? PLoS Genet 5(10): e1000694, 2009. |
| 1. Ruchat SM, Elks CE, Loos RJ, Vohl MC, Weisnagel SJ, Rankinen T, Bouchard C, Pérusse L. Evidence of interaction between type 2 diabetes susceptibility genes and dietary fat intake for adiposity and glucose homeostasis-related phenotypes. J Nutrigenet Nutrigenomics. 2(4-5):225-34, 2009. |
| 1. Gallant AR, Tremblay A, Pérusse L, Bouchard C, Després J-P, Drapeau V. The Three-Factor Eating Questionnaire and BMI in adolescents: Results from the Québec Family Study. Br J Nutr. 104(7): 1074-9, 2010. PMID: 20447324. |
| 1. Do R, Bailey SD, Paré G, Montpetit A, Desbiens K, Hudson TJ, Yusuf S, Bouchard C, Gaudet D, Pérusse L, Andad S, M-C Vohl, Pastinen T, Engert JC. Fine mapping of the insulin-induced gene 2 identifies a variant associated with LDL cholesterol and total apolipoprotein B levels. Circ Cardiovasc Genet. 3(5): 454-61, 2010. PMID: 20858904. |
| 1. Chaput JP, Sjödin AM, Astrup A, Després JP, Bouchard C, Tremblay A. Risk factors for adult overweight and obesity: the importance of looking beyond the “Big Two”. Obesity Facts 2010; 3: 320-327. |
| 1. Dolley G, Lamarche B, Després JP, Bouchard C, Pérusse L, Vohl MC. Investigation of LRP8 gene in 1p31 QTL linked to LDL peak particle diameter in the Quebec family study. Mol Genet Metab. 102(4):448-452, 2011. PMID: 21316997. |
| 1. Rhéaume C, Arsenault BJ, Dumas MP, Pérusse L, Tremblay A, Bouchard C, Poirier P, Després JP. Contributions of cardiorespiratory fitness and visceral adiposity to six-year changes in cardiometabolic risk markers in apparently healthy men and women. J Clin Endocrinol Metab. 96(5):1462-8, 2011. PMID: 21325457. |
| 1. Dolley G, Boisclair ME, Lamarche B, Després JP, Bouchard C, Pérusse L, Vohl MC. Interactions between dietary fat intake and FASN genetic variation influence LDL peak particle diameter. J Nutrigenet Nutrigenomics. 4(3):137-45, 2011. PMID: 21646813. |
| 1. Chaput JP, Després JP, Bouchard C, Tremblay A. The association between short sleep duration and weight gain is dependent on disinhibited eating behavior in adults. Sleep 2011; 34: 1291-1297. |
| 1. Kilpeläinen TO, Qi L, Brage S, …, Loos RJ. Physical activity attenuates the influence of FTO variants on obesity risk: A meta-analysis of 218,166 adults and 19,268 children. PLoS Med. 8(11):e1001116, 2011. PMID: 22069379. PMCID: PMC3206047 |
| 1. Huot M, Arsenault BJ, Gaudreault V, Poirier P, Pérusse L, Tremblay A, Bouchard C, Després JP, Rhéaume C. Insulin resistance, low cardiorespiratory fitness, and increased exercise blood pressure: contribution of abdominal obesity. Hypertension. 58(6):1036-42, 2011. PMID: 22025379. |
| 1. Choquette AC, Bouchard L, Drapeau V, Lemieux S, Tremblay A, Bouchard C, Vohl MC, Pérusse L. Association between olfactory receptor genes, eating behavior traits and adiposity: Results from the Quebec Family Study. Physiol Behav. 105(3):772-6, 2012. PMID: 22044667. |
| 1. Chaput JP, Després JP, Bouchard C, Tremblay A. Short sleep duration preferentially increases abdominal adiposity in adults: preliminary evidence. Clinical Obesity 2011; 1: 141-146. |
| 1. Manning AK, Hivert MF, Scott RA, …, Langenberg C. A genome-wide approach accounting for body mass index identifies genetic variants influencing fasting glycemic traits and insulin resistance. Nat Genet. 13;44(6):659-669, 2012. PMID: 22581228. PMCID: PMC3613127. |
| 1. Chaput JP, Després JP, Bouchard C, Tremblay A. Longer sleep duration associates with lower adiposity gain in adult short sleepers. International Journal of Obesity 2012; 36: 752-756. |
| 1. Chaput JP, McNeil J, Després JP, Bouchard C, Tremblay A. Short sleep duration is associated with greater alcohol consumption in adults. Appetite 2012; 59: 650-655. |
| 1. Gallant AR, Tremblay A, Pérusse L, Després JP, Bouchard C, Drapeau V. Past dieting is related to rigid control and disinhibition in adolescents from the Québec family study. Br J Nutr. 14;108(11):1976-9, 2012. PMID: 22369701. |
| 1. Saunders TJ, Tremblay MS, Després JP, Bouchard C, Tremblay A, Chaput JP. Sedentary behaviour, visceral fat accumulation and cardiometabolic risk in adults: a 6-year longitudinal study from the Quebec Family Study. PLoS One 2013; 8: e54225. |
| 1. Plourde M, Vohl MC, Bellis C, Carless M, Dyer T, Dolley G, Marette A, Després JP, Bouchard C, Blangero J, Pérusse L. A variant in the LRRFIP1 gene is associated with adiposity and inflammation. Obesity (Silver Spring). 21(1):185-92, 2013. PMID: 23505185. |
